# AI and social science: Automatic classification tools for big data analysis in sociological research

**DOI:** 10.1371/journal.pone.0351336

**Published:** 2026-06-18

**Authors:** Andrea Nucita, Assunta Penna, Antonia Cava, Giancarlo Iannizzotto, Massimo Mucciardi

**Affiliations:** Department of Cognitive Sciences, Psychology, Education, and Cultural Studies, University of Messina, Messina, Italy; University of Naples Federico II: Universita degli Studi di Napoli Federico II, ITALY

## Abstract

This study examines the use of Social Network Sites for public institutional communication through a sociological, data-driven lens, focusing on the challenges and potential of automated classification tools for data analysis. Although Large Language Models are increasingly used to process social media data, a key research gap remains: few studies systematically assess whether AI-based categorizations are as reliable as human coding, especially when categories are semantically ambiguous. The research addresses the following questions: How reliable are AI-generated classifications compared to those made by human experts? Is human–machine agreement comparable to the level of agreement observed among human coders? To experimentally test this approach, we conducted a case study on Facebook posts published by two Italian universities (March 2020–March 2023), classified into eight categories of public institutional communication. Three researchers independently annotated the dataset. Human annotations are used as a benchmark to assess agreement patterns and to compare them with classifications produced by AI-based systems. Results show substantial interpretive ambiguity across several categories, mirrored by variability among human coders. Nonetheless, automated models achieve agreement with human classifications that is broadly comparable to inter-coder agreement. Overall, the findings support integrating AI as an additional coder within hybrid workflows to enable scalable and transparent sociological analysis of complex social media data.

## Introduction

A sociological approach to the study of Social Network Sites (SNS), based on the quantitative nature of data (data-driven), delineates an ambivalent scenario for sociological analysis. On one hand, the distinctive characteristics of digital data, such as persistence and searchability, represent a valuable resource for researchers eager to access a vast amount of data for analysis [1]. However, the continuous massive flow of data published online at every moment can create significant obstacles for a quantitative approach. Challenges include the complexity of acquiring such data and the difficulties of managing such a large volume of information.

Large Language Models (LLMs) have demonstrated their capacity to generate and analyze large-scale text-based datasets, offering new ways to study public discourse, behavioral patterns, and policy decisions [2,3]. Despite their potential, LLMs pose significant methodological and ethical challenges. Ethical issues related to the use of LLMs in social research involve several critical aspects, including methodological transparency, data privacy, risk of bias, scientific reproducibility, and accountability of automated decisions.

While these models offer powerful linguistic capabilities [4,5], there is a need to demystify their functioning and critically assess their limitations, particularly their tendency to reflect biases embedded in training data [6,7]. Studies using LLM-generated data run the risk of misrepresentation of smaller, potentially more vulnerable populations, essentially reproducing age-old data colonialism problems of social research. LLMs, in fact, learn from vast datasets, which may reflect existing cultural, political and social biases. This can lead to distorted results. With respect to this critical issue, Rossi et al. [8] suggest that bias that derives from biased data should not be considered as a problem but as a feature of the system.

Besides, although LLMs can facilitate the analysis of large datasets, the automated extraction of information from social media and online forums, just as an example, raises ethical issues related to the protection of personal data.

Automated classification using LLMs offers a powerful tool for processing large textual datasets in social science research, but its effective integration requires a balanced approach combining human expertise and AI. This study evaluates the use of a fine-tuned BERT model to classify Facebook posts from Italian university pages, with the aim of comparing human–machine agreement with inter-coder agreement among researchers. By focusing on a high-ambiguity classification task grounded in public communication categories [9], the study addresses a key gap in the literature, namely the limited number of systematic comparisons between AI-generated and human classifications that also account for variability among human annotators.

Accordingly, this study asks: how reliable are the classifications generated by AI models when compared to those made by human experts? More specifically, is the agreement between AI-generated classifications and those produced by researchers comparable to the level of agreement observed among the researchers themselves?

## Literature review

### Social networks as platforms

According to Carrigan and Jordan [10], the specific characteristics of higher education platforms are as follows:

- Data-generating: In recent years, a substantial body of literature on “big data” has emerged, often characterized by dimensions such as volume, variety, velocity, and veracity [11]. Generated as a byproduct of user interactions with digital platforms, these data accumulate rapidly and are typically processed in real time. Their scale and speed exceed human analytical capacities, making automated methods essential for their organization and analysis.
- Opacity: While data are generated as a byproduct of user activity, platform owners maintain privileged access to them. This creates an asymmetrical structure in which user-facing interfaces coexist with backend systems designed to analyze and influence behavior. As a result, users are primarily treated as objects of prediction, with their actions reduced to data traces feeding real-time analytical processes.
- Lock-in: Platforms tend to retain users through various mechanisms. Their use entails investments of time and attention, creating cumulative sunk costs. At the same time, platforms operate through their own classificatory systems—sets of categories embedded in the interface—that shape how users interpret and respond to interactions, for example by encouraging engagement through metrics such as followers, likes, or shares.

Social network sites, characterized by visibility, accessibility, and widespread use, have become established platforms within the university system, moving from a marginal to a central role in academic communication. Despite this, the relationship between university public communication and the use of social media as institutional channels remains underexplored. Existing research (e.g., [12]) highlights a shift from descriptive accounts of informal use toward the recognition of the need for strategic communication, emphasizing audience targeting and platform-specific dissemination as key challenges.

### Social media in public communication

To understand the potential of social media in the public sphere, various scholars have conducted in-depth research. The works of Lovari [13], Lovari and Piredda [14], Ducci [15], Freberg [16], Lovari and Valentini [17], Johann et al., [18], Wigley and Zhang [19], have identified and analyzed the multiple ways of using social network site platforms and their growing importance within communication strategies increasingly integrated into public relations and communication programs. The layered presence of social media has had a significant impact on the institutional sphere, redefining communication and interaction between Public Administration and citizens.

As previously mentioned, social media platforms are particularly adopted and promoted communication channels by universities, aiming for “connectivity” of the academic community, seizing primarily the opportunity for effective promotion of the institution’s identity image and to represent a solid and distinctive reputation [20, 21, 22]. Equally significant aspects of university communication through social media emerge from a summary review of the international literature on the subject [23]: the ability to attract potential students [24], preferences regarding post formats and categories of content published on chosen social media platforms [25], and engagement in relation to factors such as the type of content published or the chosen format for social media posts [22,26,27]; the evaluation of universities’ presence on platforms [28], and the communication resources employed [20].

The breadth and specificity of investigation relevant to our research work make the study by Capriotti and Zeler [23] significant. They aim to analyze the institutional communication of universities on social media by conducting a content analysis of the communication strategies of 70 university entities in the United States, Europe, and Latin America. The study identifies and analyzes three main dimensions (posting, interactivity, and content) of institutional communication on social media in universities, broadening the understanding of social media management. The results reveal different dynamics in the management of institutional communication of universities on social media. Initially, universities were implementing institutional communication on social media characterized by limited dissemination of proprietary content. According to the authors, although with some variations between territories, this reflects trends already emerged in previous studies [20]. Secondly, a clear trend towards the increasing adoption of dialogic resources and interactive strategies emerges. However, universities maintain a predominantly informative approach in their social networks. The priority seems oriented towards institutional content over those related to teaching and research. This raises questions about the authenticity of the dialogue and the effectiveness of online communication with university users.

### Public institutional communication

In our study, we will adopt the definition of Ducci and Lovari [9], according to which “public institutional communication” represents a complex, strategic, and integrated activity concerning public goods, rights, and general interest topics by public sector organizations. This activity is based on strategies and tools for information and relationship with citizens, media, and other stakeholders, pursuing impartiality and inclusiveness to promote participation in democratic life and build trust in the collective interest. Public institutional communication, in turn, is divided into different types based on purposes and content (or areas of intervention), articulated in various sectors. The authors specifically identify eight types or sectors of public institutional communication that we will use in the empirical part dedicated to content analysis (see Table 1). It is important to note that this distinction is theoretical and may involve overlaps and hybridizations in the operational activities within the same public institutions (Ducci & Lovari, [9] pp.22–28):

**Table 1:** Description of the areas and contents of Public Institutional Communication.

1	Communication of Institutional Activities	Refers to communication characterized by the illustration of activities and tasks proper to Public Administration as an institution in its complexity/entirety and not in its individual articulations/services. It includes organizing events, participating in fairs/conferences, meetings, and interinstitutional meetings, as well as planning institutional communication campaigns.
2	Brand Communication	Involves communication processes aimed at promoting the image and caring for the reputation of the public institution, through the communication of its identity, mission, and vision. Its primary purpose is to increase the institution’s knowledge and enhance its recognizability and attractiveness to various publics. It includes activities undertaken to value and promote the identity, distinctive characteristics of the entity, and its history/achievements.
3	Normative Communication	It encompasses the communication activities with which the institutions make their acts visible to the public through a process of simplification of language, the adoption of publicity criteria, ways of searching and consulting documents, online and offline. It is a type of communication whose function is to illustrate and make comprehensible rules (laws, legislative decrees, decree-laws, regulations, etc.) of different legal rank.
4	Service Communication	This is communication focused on the services provided by the public administration, to facilitate their knowledge, access and use by citizens. It should be taken care of with an integrated service communication approach and also following the principles of marketing applied to the public sector (Fiorentini 1995). This means accompanying with communication, through multi-channel strategies, the entire life cycle of a public service: when it is born to make it known, at the time of its widespread use to maintain a high level of notoriety, in the obsolescence phase, to communicate the imminent or completed cessation of the service.
5	Communication for Participation	This is the communication that is taken care of to foster citizens’ participation in decision-making processes, with a view to participatory and shared administration (Arena 1997; Faccioli – D’Ambrosi – Massoli 2007). In fact, participatory and inclusive processes, facilitated today using advanced network technologies, need to be accompanied in the various stages of their development by adequate online and offline communication that makes them knowable and facilitates real participation by as many citizens as possible.
6	Public Policy Communication	This type of communication concerns the visibility and promotion of so-called public policies, a very broad concept that we use here with specific reference to the decisions that administrations take on how to manage public resources, the fruit of citizens’ contributions [...] In essence, public policy communication is what can be identified internationally as government communication, a communication that must be clear, easily accessible, as impartial and objective as possible, on public action, but that can also contain elements/traits of image promotion of the administration and its political component (Pasquier 2012). It is a communication that must enable citizens to know, and consequently create an opinion, about the way in which administrations use public resources.
7	Communication of socially relevant issues	The public administration has an obligation to communicate on socially relevant issues (issues) concerning various aspects such as culture, health, environment, safety, inclusion, human rights, etc. The aim is to promote ideas, attitudes, behaviors or values that are considered indispensable to promote individual and collective wellbeing at a certain moment in time. The communication campaigns that public administrations carry out on these issues are to be considered an integral part of a social marketing approach (Factors 2020; Lee – Kotler 2015; 2019), and are often associated with the promotion of a service, an activity, a norm [...] Let us add that social communication performs an important function of symbolic integration (Mancini 2002), contributing precisely to enriching the collective symbolic universe around issues of particular relevance for society as a whole, as well as fostering an anchoring function on new emerging issues (e.g., gender equality, vaccinations, digital inequalities, energy saving).
8	Internal Communication and Human Resources Development	This is a fast-growing field that takes into account the extension of the scope of internal communication, both in the academic and operational spheres, as well as the impact of the pandemic with the systematic development of remote forms of working (teleworking, agile working, etc.). The definition no longer refers only to organizational boundaries (internal employees vs. external publics), but also considers those boundary publics (suppliers, consultancy firms, etc.) that collaborate for several reasons and purposes with the administration in a non-episodic manner. These flows are not only aimed at transferring instructions and messages from the administration to collaborators (top-down) to perform tasks, duties, and activities more efficiently and effectively (Mazzei2004). Instead, it is a vision of internal communication as a set of interaction and relationship processes aimed at generating intangible resources: the knowledge needed for work processes and the active alliance of those involved so that a sense of identification with the organization, community and collaboration is developed (Mazzei2018). This vision underlies relationship processes that emphasize the centrality of active communication behaviors on the part of employees (Kim – Grunig 2011), such as seeking and sharing information and knowledge, and defending the administration in the event of attacks, emergencies, or crises (Men – Bowen 2017).

Source: Ducci & Lovari [9].

### Manual and automated post classification

As we will see in more detail in the next section, the data subject of our research were categorized by three researchers (scholars in the field of institutional public communication) and a third categorization was conducted using the davinci-002 model developed by OpenAI (More information on the models available at https://platform.openai.com/docs/models).

During the period between April 2023 and June 2023, the researchers performed a manual categorization of the content (a total of 2487 posts from the two universities), assigning the information in the posts to categories identified according to criteria outlined in the literature [9].

This phase was characterized by a separate process in which the researchers applied their experience in the field of institutional public communication to interpret and assign the information to the appropriate categories. Subsequently, to assess the classification capacity of an automated system, the post texts were subjected to classification using the OpenAI API, employing the davinci-002 model. The combination of the manual approach of the researchers with the classification capabilities of the automatic model may provide interesting insights into the opportunities and criticalities of using artificial languages in social research.

Several studies have explored how LLM-based classification can be embedded into a researcher’s workflow to enhance both speed and rigor. LLMs can automatically classify and filter large text datasets, helping researchers identify relevant content before manual review [29]. LLMs can conduct inductive thematic analysis, generating preliminary topic clusters that guide deeper human interpretation [30]. Researchers can use LLMs in an iterative feedback loop, where initial classifications are manually adjusted, and the refined categories are fed back into the model for improved accuracy [31].

Very few studies have compared classifications generated by artificial intelligence models with human classifications [32,33], our research aims to make a contribution in this direction.

## Materials and methods

### Data and classification task metrics

The data used for the analysis consisted of posts from the official Facebook pages of two Italian universities: the University of Messina and the University of Salerno. Specifically, the dataset included a total of 1,030 posts from the University of Messina and 1,459 posts from the University of Salerno. The posts were collected between March 2020 and March 2023 in compliance with Meta’s data usage policies. The text of the posts was extracted directly from the Facebook pages of the universities by parsing the page content saved through the browser. No data cleaning intervention was conducted on the posts. The texts were analyzed in their original form, including all emojis, links, hashtags, and other elements as they appeared on the Facebook posts. The classification of the posts was based on eight predefined categories. While the raw data cannot be shared due to Meta’s data usage policies, the data produced by the authors — including the annotated datasets — are publicly available on Hugging Face (https://huggingface.co/HuM-HILab/pub_comm_classification).

These categories were described in the preceding section and are outlined in Table 2.

**Table 2:** Classification categories.

Category code	Category label
0	Communication for Participation
1	Communication of Institutional Activities
2	Communication of socially relevant issues
3	Public Policy Communication
4	Brand Communication
5	Service Communication
6	Internal Communication and Human Resources Development
7	Normative Communication

The manual classification process involved three researchers (here named: R1, R2, and R3). They are all specialized in Communication Studies and share a common background in the literature on public communication, particularly in the Italian context. Each researcher independently classified the posts without sharing their results with the others, thus avoiding mutual influence among evaluators. Before analyzing the capability of an automatic system to classify the posts, we first evaluated the agreement among individual researchers. For this purpose, to assess the reliability of agreement among a fixed number of human raters, we selected Fleiss’ Kappa (κ) [34], which is specifically designed for measuring inter-rater reliability in multi-rater settings and for categorical classification tasks. Unlike Cohen’s Kappa, which is limited to the assessment of agreement between two raters, Fleiss’ Kappa allows for the simultaneous evaluation of agreement among more than two evaluators. This measure was chosen because it accounts for agreement occurring by chance and provides a standardized way to compare the consistency of annotations across multiple evaluators.

To calculate Fleiss’ Kappa (κ), the following formula was used:


$$\begin{array}{c} \kappa =\frac{p_{o}-p_{e}}{\text{1}-p_{e}} \end{array}$$

where $p_{o}$ is the observed proportion of agreement and $p_{e}$ is the proportion of agreement expected by chance.

This metric allows us to quantify inter-rater agreement: a value of 0 indicates agreement equivalent to chance, while a value of 1 indicates complete agreement. Landis and Koch [35] provide interpretations for intermediate values, as shown in Table 3.

**Table 3:** Landis and Koch (1977) k value explanation.

below 0.0 Poor
0.00–0.20 Slight
0.21–0.40 Fair
0.41–0.60 Moderate
0.61–0.80 Substantial
0.81–1.00 Almost perfect

### Methodology

#### Step 1.

The aim of the first step is to investigate the feasibility of using AI-based automated classification in comparison with, or as a complement to, human classification performed by researchers. This step includes two key components:

1. assessing the degree of agreement among the researchers themselves, and
2. evaluating how closely a zero-shot classification model aligns with those human judgments.

The first component is essential, as human classifications are not perfectly consistent—different researchers may apply the same classification schema with subtle variations. Therefore, measuring inter-rater agreement provides a necessary baseline for interpreting the validity of any comparison with AI outputs.

The second component involves introducing a zero-shot classification model, specifically the davinci-002 model accessed via the OpenAI API. This choice was motivated by the model’s high accuracy in text understanding and classification tasks. Its balance between performance and cost makes it suitable for large-scale experimentation. This step aimed to evaluate the reliability of a Large Language Model (LLM) [36] in a zero-shot classification setting [37]. Zero-shot classification allows a model to assign categories to data without having been explicitly trained on labeled examples of those categories.

**Fig 1:**
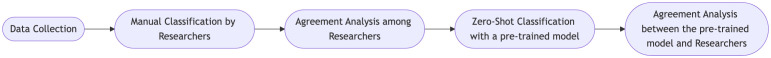
Methodological workflow for Step 1: evaluation of the feasibility of zero-shot classification. The process includes manual classification by researchers, assessment of inter-rater agreement, agreement analysis with a zero-shot approach of a pre-trained model.

The full methodological workflow for Step 1 is shown in Fig 1. It includes the manual classification of Facebook posts by three researchers, the measurement of inter-rater agreement, and the comparison of those results with the output of the zero-shot classification performed by the LLM.

#### Step 2.

**Fig 2:**
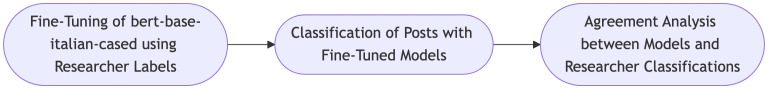
Methodological workflow for Step 2: improving automated classification through fine-tuning. The process involves training a BERT model on researcher-provided labels, using the fine-tuned models to classify posts, and evaluating agreement between the model predictions and the original human classifications.

In the second phase of the study, the goal was to improve the accuracy and alignment of automated classification by fine-tuning a language model using human-annotated data. Specifically, a BERT model was fine-tuned on various combinations of labels provided by different researchers, reflecting different interpretations of the classification criteria. These customized models were then used to classify the same set of Facebook posts. Finally, the outputs of the fine-tuned models were compared with the original human classifications to assess their level of agreement and evaluate whether fine-tuning improved consistency with researcher judgments. Fig 2 shows schematically the process.

To enhance the automatic classification and create a model specifically trained with the researchers’ classification data, we decided to apply a Bidirectional Encoder Representations from Transformers (BERT) model pre-trained in Italian (More information about the model is available here: https://huggingface.co/dbmdz/bert-base-italian-cased). This model was fine-tuned using the researchers’ classification results. BERT models are particularly well-suited for text classification tasks for several reasons. Since their introduction [38], and have been proved to be among the most performant Large Language Models (LLMs) in the literature. BERT models can be fine-tuned on task-specific data, enabling adaptation to a specific analytical context and alignment with researcher-defined classification criteria.

## Analyses‌‌

### Research question1

**Fig 3:**
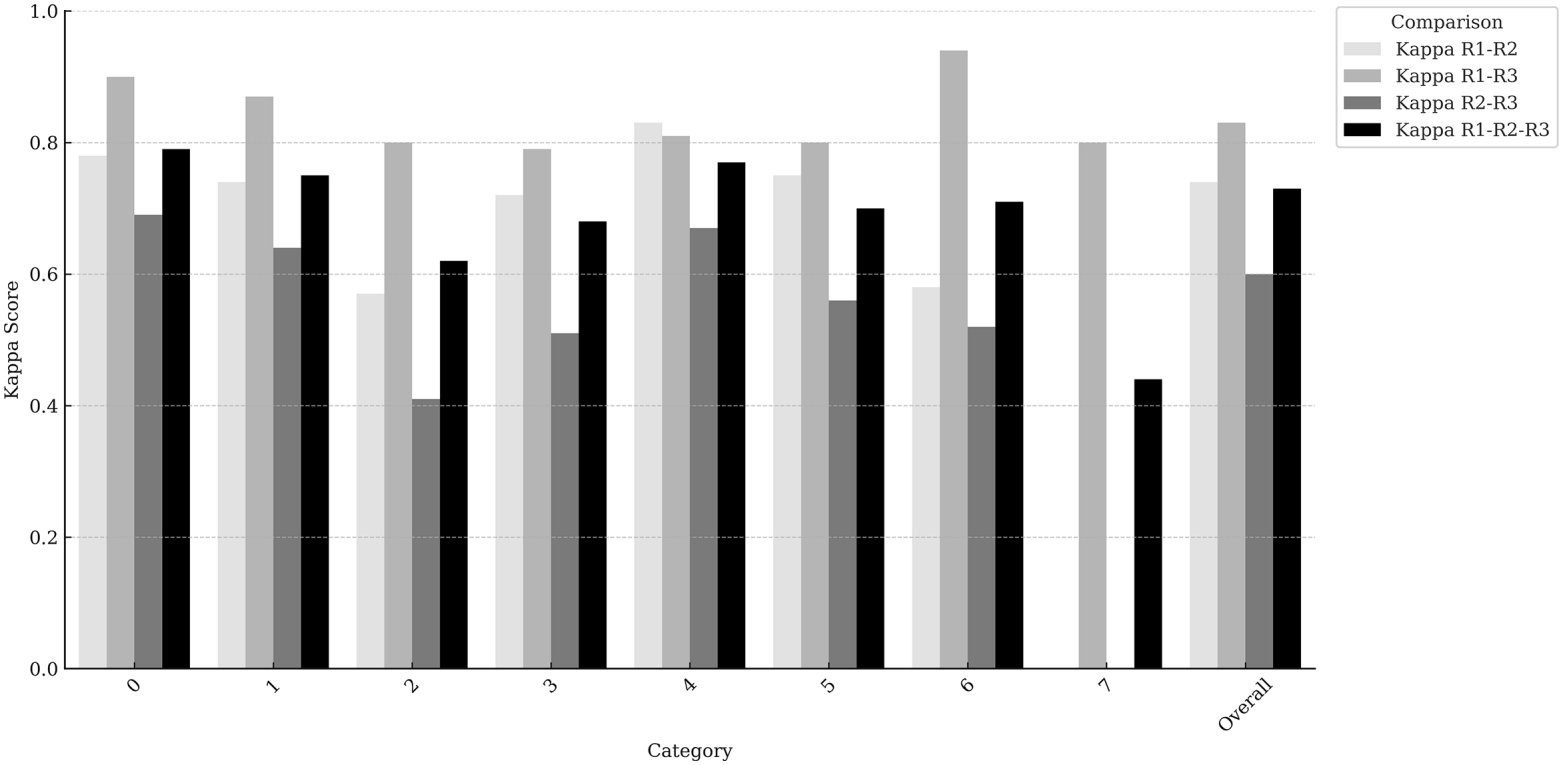
Fleiss k values, comparing researchers’ classifications. R1-R2 combines classification data randomly 50% from R1 and R2, in the same way for R2-R2; R1-R2-R3 combines ⅓ of data from the three researchers.

Despite sharing a common background, the researchers exhibited moderate agreement in their classifications. This observation evidences the inherent subjectivity in human text classification. Below are the detailed Fleiss’ Kappa results, as Fig 3 shows.

These results underscore the variability in agreement among researchers, even within the same domain. While some categories show high levels of agreement, others reveal substantial differences in interpretation. The overall kappa values suggest that while there is a reasonable degree of consensus with R1 and R2, R1-R3 and R1-R2-R3, the couple R2-R3 shows a lower agreement.

This variability points to the challenges inherent in human-made classification and highlights the potential value of developing robust automated systems to support or enhance consistency in classification tasks.

A central challenge in both human and machine-based classification lies in semantic ambiguity. Many social media messages are inherently open to multiple interpretations due to factors such as polysemy, implicit tone, or overlapping communicative functions (e.g., in our categories Communication of Institutional Activities and Normative Communication). This often leads to disagreement among human annotators, even when they are trained experts. In such contexts, classification is not a matter of objectively “correct” labelling, but rather of interpretive judgment.

The same posts were subsequently classified using the OpenAI API, specifically utilizing the davinci-002 model, as described above.

**Fig 4:**
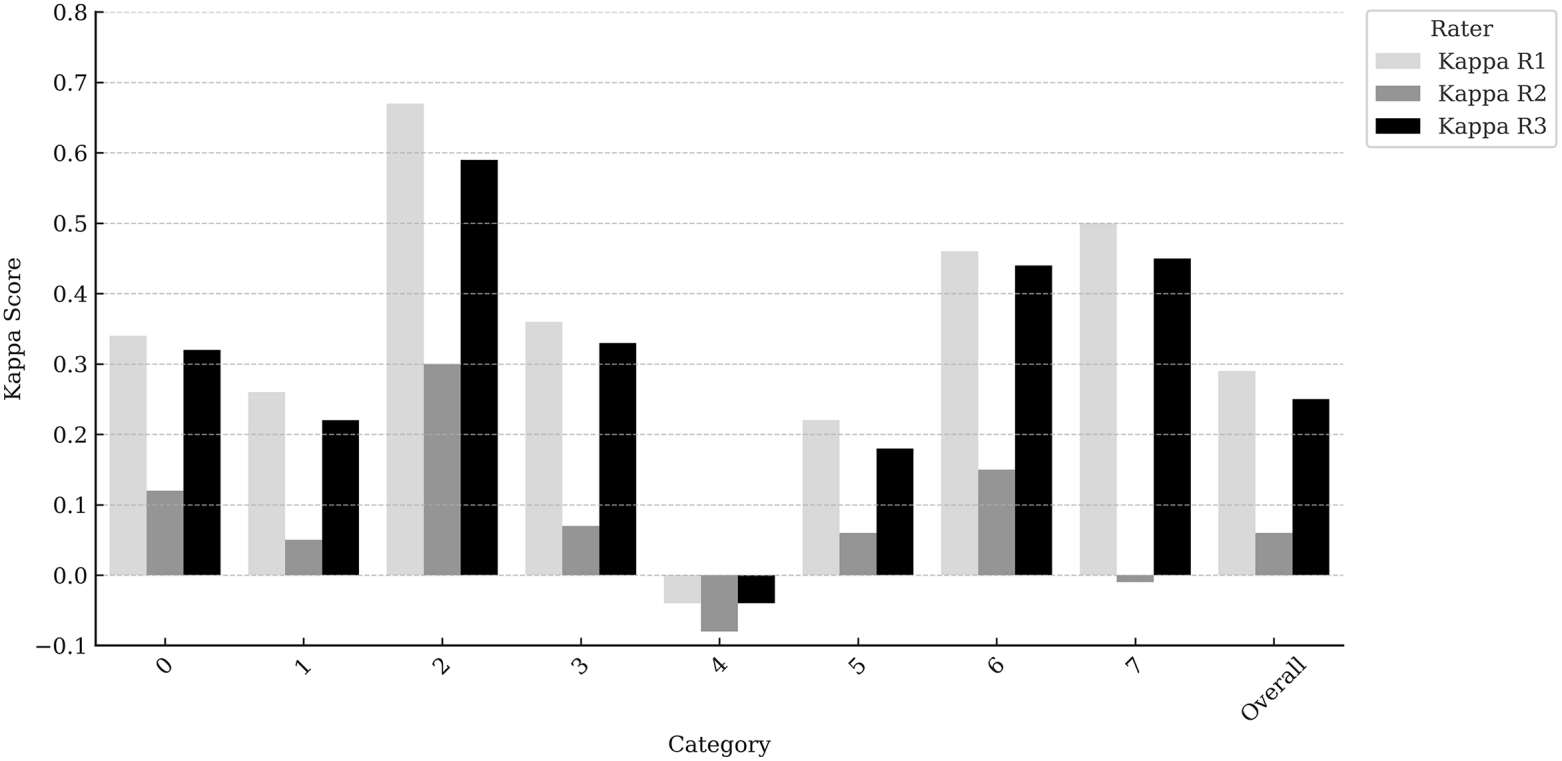
Kappa index for overall agreement and for each category.

We compared the classification results obtained from OpenAI’s API with those provided by the three researchers. The results are summarized in Fig 4, where Fleiss’ Kappa values are calculated to measure the agreement between the classifications. Although the overall Fleiss’ Kappa values are not particularly high, indicating moderate agreement, the results reveal a certain degree of agreement in classifications across various categories. Some categories show a moderate or substantial level of agreement, even with a zero-shot approach.

This suggests that with further refinement and training, automated classification systems could potentially reach an agreement level that is comparable to that of human researchers, especially in categories where higher Kappa values were observed.

### Research question 2

The central question of our research is whether the level of agreement between the classifications produced by the BERT model and those generated by the researchers is comparable to the agreement among the researchers. To analyze the agreement levels in an unbiased manner, we fine-tuned the BERT model with classification samples from individual researchers as well as with combinations of classifications from different researchers.

To train and evaluate the classification models, the dataset was randomly divided using an 80/20 holdout strategy: 80% of the data was used for training, while the remaining 20% was set aside for testing. The split was performed using a fixed random seed to ensure reproducibility and to prevent any data leakage between training and evaluation phases. Each post was assigned exclusively to either the training or test set, ensuring that the model was evaluated only on unseen data.

The training data consisted of manual classifications performed independently by three researchers, each with expertise in communication studies. Separate models were trained using the full set of annotations from each individual researcher, as well as using combined datasets created by merging randomly sampled annotations from multiple researchers (e.g., 50/50 or 1/3 splits). This strategy allowed us to assess not only the effectiveness of individual annotation-based models, but also how combining different human perspectives impacted model performance and generalizability.

Inter-rater disagreement was not resolved through adjudication or consensus; instead, it was treated as an analytical variable. This allowed us to compare AI-generated classifications with human annotations, as well as agreement levels among the human annotators. Table 4 summarizes the various training datasets used, together with training metrics for each model:

**Table 4:** Model performance. F1 Micro measures overall model performance by aggregating true positives, false positives, and false negatives across all classes—giving equal weight to each instance. F1 Macro calculates the F1 score for each class independently and then averages them—treating all classes equally, regardless of their size. F1 Weighted also averages F1 scores across classes, but weights them according to the number of true instances per class—balancing class importance based on their frequency.

Model	Training Data	Accuracy	F1 micro	F1 macro	F1 weighted
**BERT_R1**	All categorized data from researcher R1	0.68	0.68	0.55	0.67
**BERT_R2**	All categorized data from researcher R2	0.71	0.71	0.56	0.71
**BERT_R3**	All categorized data from researcher R3	0.54	0.54	0.45	0.54
**BERT_R1-R2**	50% random sample from R1 and 50% from R2	0.63	0.63	0.43	0.61
**BERT_R1-R3**	50% random sample from R1 and 50% from R3	0.60	0.60	0.43	0.59
**BERT_R2-R3**	50% random sample from R2 and 50% from R3	0.64	0.64	0.44	0.62
**BERT_R1-R2-R3**	1/3 random sample from R1, R2, and R3	0.64	0.64	0.45	0.62

## Results

Each of these fine-tuned models was used to classify the full set of Facebook posts. We then compared each model’s classifications with those of the individual researchers to assess the agreement. The most insightful comparisons involved models trained on the classifications of certain researchers being compared with the classifications of researchers whose data was not used for training. For instance, BERT_R1 vs. R2, that is, comparing the model trained on R1’s classifications with R2’s classifications, or BERT_R1-R2 vs. R3, that is, comparing the model trained on a mix of R1 and R2’s classifications with R3’s classifications. The primary focus was on understanding how well the BERT model could generalize the classification task by leveraging the diversity and expertise of different researchers. By comparing the agreement levels across various combinations, we aimed to determine whether the automated system could match or exceed human agreement levels. These analyses are crucial in validating the effectiveness of fine-tuning BERT models with diverse classification data, ultimately aiming to create an automated classification system that can replicate human-level agreement and consistency. This approach not only could reduce the workload for researchers but also enhances the scalability and reliability of text classification tasks.

Table 5 presents the Fleiss’ Kappa values comparing the BERT model classifications with those of the researchers (R1, R2, and R3). The bold values highlight the comparisons where the models were evaluated against data from researchers whose classifications were not used for training the models.

**Table 5:** Fleiss k values, comparing BERT models classifications with researchers’ combined classifications. Bold values highlight the comparisons where the models were evaluated against data from researchers whose classifications were not used for training the models.

Model	R1	R2	R3	R1-R2	R1-R3	R2-R3	R1-R2-R3
**BERT_R1**	0.83	**0.70**	**0.67**	0.77	0.75	**0.69**	0.76
**BERT_R2**	**0.67**	0.88	**0.53**	0.76	**0.60**	0.71	0.65
**BERT_R3**	**0.67**	**0.60**	0.68	**0.63**	0.68	0.65	0.67
**BERT_R1-R2**	0.73	0.79	**0.59**	0.82	0.67	0.64	0.71
**BERT_R1-R3**	0.77	**0.69**	0.69	0.73	0.77	0.73	0.76
**BERT_R2-R3**	**0.69**	0.77	0.64	0.67	0.71	0.81	0.70
**BERT_R1-R2-R3**	0.76	0.70	0.68	0.76	0.75	0.70	0.80

The BERT_R1 model shows the highest agreement with R1, which was expected as the model in this case was trained on R1’s classifications. Interestingly, the model also shows a good degree of agreement with the combined classifications, especially with R1-R2 and R1-R3, indicating that the model trained on R1’s data can generalize reasonably well to combinations involving R1. The agreement with R2-R3 is moderate (0.69), suggesting some consistency but also indicating areas where the model might diverge. The BERT_R2 model, as expected, shows the highest agreement with R2. When compared with R1 (0.67) and R3 (0.53), the agreement is lower, highlighting the unique classification patterns of R2 that are not fully captured by R1 or R3. The moderate agreement with the R1-R3 combination (0.60) and the overall combination (0.65) shows that the model trained on R2’s data does not generalize as well as BERT_R1, particularly when R3’s classifications are involved. The BERT_R3 model shows the highest agreement with R3. The agreement levels with R1 (0.67) and R2 (0.60) are moderate, indicating some level of shared understanding but also notable differences. The model’s performance with the combined datasets (0.63 with R1-R2, 0.68 with R1-R3, and 0.65 with R2-R3) indicates that the model can integrate information from multiple sources but has limitations in aligning perfectly with all combinations.

## Discussion

This study investigates the use of automated classification tools in sociological research, focusing on the analysis of large datasets from social media. The case study involves public institutional communication on Facebook by two Italian universities. The researchers compared manual classification by multiple human coders with automated classifications generated by AI models.

The analyses conducted aimed to investigate how a specifically trained AI model can assist researchers in a complex and demanding task such as classifying a large volume of social media data. To achieve this, we compared the classifications made by multiple researchers on the same data with those made by AI models against the researchers’ classifications.

The proposed case study is particularly complex, as the text needs to be classified into eight categories, some of which are ambiguous. This ambiguity is reflected in the differing performance of the models across various categories. For instance, Researcher 2 did not use category 7 at all, as she found none of the posts fit into that category.

However, when we compare the Fleiss’ Kappa index values in the eight categories of the trained models with those that were not trained by the researchers, we observe that, on average, the values are higher and exhibit less variation among the categories (see Table 6).

**Table 6:** Kappa index for each category for BERT Models with Researchers’ Pre-Training.

Category	BERT_R1	BERT_R2	BERT_R3	
0	0.749	0.916	0.632	
1	0.910	0.940	0.816	
2	0.821	0.731	0.574	
3	0.770	0.822	0.631	
4	0.906	0.933	0.812	
5	0.873	0.919	0.759	
6	0.713	0.552	0.389	
7	0.320	–	0.370	

Nevertheless, based on the findings from the previous section and as illustrated in Table 5, the Fleiss’ Kappa values suggest a substantial agreement between the BERT models and human-made classifications, reflecting similar results to those described in Fig 3, where the classifications of the three researchers were compared.

Moreover, the combination of classifications among various researchers does not significantly improve the agreement of the models with the data not involved in the training. This suggests that the variability observed among the researchers is understandably reflected in the models trained with their classifications. Despite combining the classification data, the inherent differences in individual classification approaches persist, influencing the model’s performance.

These findings highlight the complexity of manual classification tasks and the potential of fine-tuned models to achieve comparable levels of agreement with human classifiers.

This is a promising indication of the viability of automated tools in complex sociological tasks.

From a methodological and epistemological perspective, our analysis is grounded in the distinction between two complementary evaluation dimensions. The first concerns model accuracy, which is primarily related to the fine-tuning process and can be interpreted as a technical performance measure, reflecting how effectively the model learns from labeled data. The second dimension concerns the degree of agreement, both among human researchers and between researchers and AI models. In this case, misalignment should not be interpreted as a failure of the model, but rather as an informative result.

The introduction of an AI model is not intended to impose an objective or definitive interpretive framework—something that is neither feasible nor desirable in the context of semantically ambiguous sociological data. Instead, the goal is for the model to reproduce a level of variability comparable to that observed among human coders. In this sense, disagreement is not an error to be eliminated, but a characteristic of the classification task itself.

An AI model can therefore be considered suitable for large-scale data processing insofar as it does not diverge from human classifications in a substantially different way than researchers already diverge from one another. Under this perspective, fine-tuned models do not replace human interpretation, but align with it, functioning as scalable extensions of human classificatory practices rather than as arbiters of a single “correct” classification.

## Limitations

This study presents several limitations that should be acknowledged.

First, the high degree of semantic ambiguity across certain categories—reflected in both inter-coder variability and uneven model performance—indicates that content classification remains an inherently interpretive process. This challenges the assumption that machine-based classification can achieve full objectivity or consistency, even when trained on human-labeled data.

Second, AI models do not eliminate disagreement but rather reproduce patterns of variability present in human annotations. As a result, their outputs should be interpreted as aligned with, rather than independent from, human judgment.

Third, the empirical context of the study—focused on Facebook posts from Italian universities—may limit the generalizability of the findings. However, the primary aim of the research is methodological rather than domain-specific. For this reason, the relevance of the results lies less in the specific dataset and more in the proposed approach to evaluating human–machine agreement in complex classification tasks.

From a methodological perspective, these findings suggest that AI tools are best understood as “additional coders” rather than substitutes for human interpretation. While they can enhance efficiency in large-scale analyses, their use should be embedded within hybrid workflows that preserve human oversight.

## Conclusion

In conclusion, this study advances methodology by evaluating not only the performance of automated classification models but also the level of agreement among multiple researchers. For future sociological research, this study points to two key directions. First, efforts should be made to refine classification schemes by testing their clarity and interpretive stability before applying them at scale—whether manually or through AI. Second, researchers must develop more transparent validation protocols to assess not only model performance, but also the epistemological assumptions embedded in the classification process.

Finally, as pointed out in the introduction, while LLMs present transformative opportunities for social science research, their integration requires a critical and ethical approach. It is necessary to focus on improving transparency, mitigating biases, and developing robust validation frameworks to ensure that these models contribute meaningfully to the field of research. A hybrid methodological approach, combining human expertise with LLM-assisted analysis, appears to be the most promising path forward.

## References

[1] Rossi L, Di Lascio FML, Pacelli B, Magnani M. Descrivere un social network site: un approccio empirico. In: Boccia Artieri G, editor. Gli effetti sociali del web. Forme della comunicazione e metodologie della ricerca online. Franco Angeli; 2015. p. 52–62.

[2] Grossmann I, Feinberg M, Parker DC, Christakis NA, Tetlock PE, Cunningham WA. AI and the transformation of social science research. Science. 2023;380(6650):1108–9. doi: 10.1126/science.adi1778 37319216

[3] Gürcan Ö. LLM-augmented agent-based modelling for social simulations: challenges and opportunities. In: HHAI 2024: Hybrid Human AI Systems for the Social Good. 2024. p. 134–44.

[4] Yang Y, Duan H, Liu J, Tam KY. LLM-Measure: generating valid, consistent, and reproducible text-based measures for social science research. 2024. https://arxiv.org/abs/2409.12722

[5] Kmainasi MB, Shahroor AE, Hasanain M, Laskar SR, Hassan N, Alam F. LlamaLens: specialized multilingual LLM for analyzing news and social media content. 2024. https://arxiv.org/abs/2410.15308

[6] Valdenegro D. A LLM digest for social scientist. SocArXiv preprint. 2023. p. 1–11.

[7] Fang X, Che S, Mao M, Zhang H, Zhao M, Zhao X. Bias of AI-generated content: an examination of news produced by large language models. Sci Rep. 2024;14(1):5224. doi: 10.1038/s41598-024-55686-2 38433238 PMC10909834

[8] Rossi L, Harrison K, Shklovski I. The problems of LLM-generated data in social science research. Sociologica. 2024;18(2):145–68.

[9] Ducci G, Lovari A. L’evoluzione della cultura della comunicazione pubblica in Italia. In: Paltrinieri R, Spillare S, Tardivo G, editors. Orizzonti Medi-terranei. Comunicazione, istituzioni e prospettive mediatiche in un confronto tra Italia e Spagna. Franco Angeli; 2022. p. 17–32.

[10] Carrigan M, Jordan K. Platforms and Institutions in the Post-Pandemic University: a Case Study of Social Media and the Impact Agenda. Postdigit Sci Educ. 2022;4(2):354–72. doi: 10.1007/s42438-021-00269-x 40477436 PMC8566191

[11] Carrigan M. Social media for academics. Sage; 2019.

[12] Coleman BC, Pettit SK, Buning MM. Social media use in higher education: do members of the academy recognize any advantages? J Soc Media Soc. 2018;7(1):420–42.

[13] Lovari A. Networked citizens. Comunicazione pubblica e amministrazioni digitali. Franco Angeli; 2013.

[14] Lovari A, Piredda A. Comunicazione pubblica digitale. Modelli e pratiche comunicative tra amministrazioni pubbliche, media e cittadini. In: Masini M, Pasquini J, Segreto G, editors. Marketing e comunicazione. Hoepli; 2017. p. 369–91.

[15] Ducci G. Relazionalità consapevole. La comunicazione pubblica nella società connessa. Franco Angeli; 2017.

[16] Freberg K. Social media for strategic communication: Creative strategies and research-based applications. Sage Publications; 2021.

[17] Lovari A, Valentini C. Public sector communication and social media: opportunities and limits of current policies, activities, and practices. In: Luoma-Aho V, Canel MJ, editors. The handbook of public sector communication. Wiley; 2020. p. 315–28.

[18] Johann M, Wolf C, Godulla A. Managing relationships on Facebook: A long-term analysis of leading companies in Germany. Public Relat Rev. 2021;47(3):102044. doi: 10.1016/j.pubrev.2021.102044

[19] Wigley S, Zhang W. A study of PR practitioners’ use of social media in crisis planning. Public Relat J. 2011;5(3):1–16.

[20] Peruta A, Shields AB. Social media in higher education: understanding how colleges and universities use Facebook. J Mark High Educ. 2017;27(1):131–43.

[21] Zadeh A, Sharda R. How can our tweets go viral? Point-process modelling of brand content. Inf Manag. 2022;59(2):103594.

[22] Fähnrich B, Vogelgesang J, Scharkow M. Evaluating universities’ strategic online communication: how do Shanghai Ranking’s top 50 universities grow stakeholder engagement with Facebook posts? JCOM. 2020;24(3):265–83. doi: 10.1108/jcom-06-2019-0090

[23] Capriotti P, Zeler I. Analysing effective social media communication in higher education institutions. Humanit Soc Sci Commun. 2023;10(1):1–13.

[24] Hesel RA. The influence of social media sites on the college search process. studentPOLL. Art & Science Group; 2013.

[25] Cismaru D-M, Ciochina R-S, Burnei I. Trends in the online communication of universities: Social media content strategies used in higher education in Europe. In: INTED Proceedings, IATED. 2023. p. 5990–5.

[26] Lund B. Universities engaging social media users: an investigation of quantitative relationships between universities’ Facebook followers/interactions and university attributes. J Mark High Educ. 2019;29(2):251–67.

[27] Marino V, Lo Presti L. Approaches to university public engagement in the online environment: insights from Anglo-Saxon higher education. Int J Educ Manag. 2018;32(5):734–48.

[28] García GM. Universidad y medios sociales. Gestión de la comunicación en la universidad española. Rev Prisma Soc. 2018;22:20–36.

[29] Rogers R, Zhang X. The Russia–Ukraine War in Chinese Social Media: LLM Analysis Yields a Bias Toward Neutrality. Soc Media Soc. 2024;10(2). doi: 10.1177/20563051241254379

[30] De Paoli S. Performing an inductive thematic analysis of semi-structured interviews with a large language model: an exploration and provocation on the limits of the approach. Soc Sci Comput Rev. 2024;42(4):997–1019.

[31] Jiao J, Afroogh S, Xu Y, Phillips C. Navigating LLM ethics: advancements, challenges, and future directions. arXiv preprint. 2024. doi: arXiv:2406.18841

[32] Schmidt A, Wiegand M. A Survey on Hate Speech Detection using Natural Language Processing. In: Proceedings of the Fifth International Workshop on Natural Language Processing for Social Media, Association for Computational Linguistics. 2017. p. 1–10.

[33] Gorwa R, Binns R, Katzenbach C. Algorithmic content moderation: Technical and political challenges in the automation of platform governance. Big Data Society. 2020;7(1):205395171989794. doi: 10.1177/2053951719897945

[34] Fleiss JL. Measuring nominal scale agreement among many raters. Psychol Bull. 1971;76(5):378–82. doi: 10.1037/h0031619

[35] Landis JR, Koch GG. The measurement of observer agreement for categorical data. Biometrics. 1977;33(1):159–74. doi: 10.2307/2529310 843571

[36] Mani G, Namomsa GB. Large Language Models (LLMs): Representation Matters, Low-Resource Languages and Multi-Modal Architecture. In: 2023 IEEE AFRICON. IEEE; 2023. p. 1–6.

[37] Makridakis S, Petropoulos F, Kang Y. Large Language Models: Their Success and Impact. Forecasting. 2023;5(3):536–49. doi: 10.3390/forecast5030030

[38] Devlin J, Chang M, Lee K, Toutanova K. BERT: pre-training of deep bidirectional transformers for language understanding. In: Proceedings of the 2019 Conference of the North American Chapter of the Association for Computational Linguistics: Human Language Technologies. Vol 1. 2019. p. 4171–86.

