# Supplementary material for: AI and social science: Automatic classification tools for big data analysis in sociological research
Source: PLoS One. 2026 Jun 18;21(6):e0351336. doi: 10.1371/journal.pone.0351336 (PMC13278443; doi:10.1371/journal.pone.0351336)
Supplement: Attachment — Submitted filename: Response to reviewers041125.docx [file pone.0351336.s002.docx]

Journal Requirements:

When submitting your revision, we need you to address these additional requirements.

1. Please ensure that your manuscript meets PLOS ONE's style requirements, including those for file naming. The PLOS ONE style templates can be found at https://journals.plos.org/plosone/s/file?id=wjVg/PLOSOne_formatting_sample_main_body.pdf and https://journals.plos.org/plosone/s/file?id=ba62/PLOSOne_formatting_sample_title_authors_affiliations.pdf

**Response:**
Thank you, all the files have been uploaded according to the instructions.

2. In your Methods section, please include additional information about your dataset and ensure that you have included a statement specifying whether the collection and analysis method complied with the terms and conditions for the source of the data.

**Response:**

The dataset consists of public posts published by official Facebook pages of two Italian universities University of Messina and University of Salerno). Although the data were publicly accessible at the time of collection (and still remain available), Meta’s Terms of Use prohibit the redistribution of scraped content. Therefore, the dataset cannot be shared openly. The authors ensure that all data were used solely for non-commercial academic research purposes and analyzed in compliance with ethical research standards.

Instead, the datasets created by the researchers for model training have been made publicly available, along with the models, on our Hugging Face page. The link to the page is as follows: <https://huggingface.co/HuM-HILab/pub_comm_classification>. These datasets are intended to serve as a reference for the data structure and annotation format used in the study; they do not include the original text of the Facebook posts, which cannot be shared due to Meta’s restrictions.

We added the following sentence in the Methods section:

“While the raw data cannot be shared due to Meta’s data usage policies, the data produced by the authors — including the annotated datasets — are publicly available on Hugging Face (https://huggingface.co/HuM-HILab/pub_comm_classification).”

3. Please note that PLOS ONE has specific guidelines on code sharing for submissions in which author-generated code underpins the findings in the manuscript. In these cases, we expect all author-generated code to be made available without restrictions upon publication of the work. Please review our guidelines at https://journals.plos.org/plosone/s/materials-and-software-sharing#loc-sharing-code and ensure that your code is shared in a way that follows best practice and facilitates reproducibility and reuse.

**Response:**

The code employed for model training, together with the training datasets, is publicly available on our Hugging Face page (<https://huggingface.co/HuM-HILab/pub_comm_classification>). These datasets are intended to serve as a reference for the data structure and annotation format used in the study; they do not include the original text of the Facebook posts, which cannot be shared due to Meta’s restrictions.

4. We note that you have indicated that there are restrictions to data sharing for this study. PLOS only allows data to be available upon request if there are legal or ethical restrictions on sharing data publicly. For more information on unacceptable data access restrictions, please see http://journals.plos.org/plosone/s/data-availability#loc-unacceptable-data-access-restrictions.

Before we proceed with your manuscript, please address the following prompts:

a) If there are ethical or legal restrictions on sharing a de-identified data set, please explain them in detail (e.g., data contain potentially identifying or sensitive patient information, data are owned by a third-party organization, etc.) and who has imposed them (e.g., a Research Ethics Committee or Institutional Review Board, etc.). Please also provide contact information for a data access committee, ethics committee, or other institutional body to which data requests may be sent.

b) If there are no restrictions, please upload the minimal anonymized data set necessary to replicate your study findings to a stable, public repository and provide us with the relevant URLs, DOIs, or accession numbers. For a list of recommended repositories, please see
https://journals.plos.org/plosone/s/recommended-repositories. You also have the option of uploading the data as Supporting Information files, but we would recommend depositing data directly to a data repository if possible.

We will update your Data Availability statement on your behalf to reflect the information you provide.

**Response:**

The data used in this study were collected through web scraping of public Facebook pages belonging to the University of Messina and the University of Salerno, in order to analyze real-world institutional communication. Due to Meta’s data usage policies, these raw data cannot be publicly shared.

The annotations produced by the researchers (i.e., the manual classification of the posts), which were used for fine-tuning the language models, have been made openly available on Hugging Face, along with the trained models and training code: <https://huggingface.co/HuM-HILab/pub_comm_classification>. These datasets are intended to serve as a reference for the data structure and annotation format used in the study; they do not include the original text of the Facebook posts, which cannot be shared due to Meta’s restrictions.

This should be covered under the Third-party data section on <https://journals.plos.org/plosone/s/data-availability>.

5. Please provide a complete Data Availability Statement in the submission form, ensuring you include all necessary access information or a reason for why you are unable to make your data freely accessible. If your research concerns only data provided within your submission, please write "All data are in the manuscript and/or supporting information files" as your Data Availability Statement.

**Response:**

In this case, we collected data from <https://www.facebook.com/messinauniversity> and <https://www.facebook.com/unisalerno>. For this reason, the authors are unable to make the raw dataset publicly available. Researchers interested in understanding how these original posts were used in the research can contact the authors or the HuM-HI Laboratory at humhilab@unime.it or via the website humhilab.unime.it.

6. We note you have included a table to which you do not refer in the text of your manuscript. Please ensure that you refer to Table 2, 3, 4, in your text; if accepted, production will need this reference to link the reader to the Table.

**Response:**

Thank you for pointing that out. We have included specific references for Tables 2–4.

Comments to the Author

1. Is the manuscript technically sound, and do the data support the conclusions?

The manuscript must describe a technically sound piece of scientific research with data that supports the conclusions. Experiments must have been conducted rigorously, with appropriate controls, replication, and sample sizes. The conclusions must be drawn appropriately based on the data presented.

Reviewer #1: Yes

Reviewer #2: Partly

2. Has the statistical analysis been performed appropriately and rigorously?

Reviewer #1: Yes

Reviewer #2: Yes

3. Have the authors made all data underlying the findings in their manuscript fully available?

The [PLOS Data policy](https://eur01.safelinks.protection.outlook.com/?url=http%3A%2F%2Ftrack.editorialmanager.com%2FCL0%2Fhttp%3A%252F%252Fwww.plosone.org%252Fstatic%252Fpolicies.action%2523sharing%2F1%2F010f019723bc9035-292039d6-ee4c-4174-ad4b-9ee1d7cbcb77-000000%2FY3-GPZhQzMxOqPnOjCpYfQ23ZPXeiqgwrS4yRfONbQc%3D213&data=05%7C02%7Canucita%40unime.it%7C3b041f7ee59144b863f408dd9fd9ee3b%7C84679d4583464e238c84a7304edba77f%7C0%7C0%7C638842480836534990%7CUnknown%7CTWFpbGZsb3d8eyJFbXB0eU1hcGkiOnRydWUsIlYiOiIwLjAuMDAwMCIsIlAiOiJXaW4zMiIsIkFOIjoiTWFpbCIsIldUIjoyfQ%3D%3D%7C0%7C%7C%7C&sdata=ulJmWeq7%2FeiGjcVX8g2VyYYucvUpl%2BKlSIe66Et%2BOQ8%3D&reserved=0) requires authors to make all data underlying the findings described in their manuscript fully available without restriction, with rare exception (please refer to the Data Availability Statement in the manuscript PDF file). The data should be provided as part of the manuscript or its supporting information, or deposited to a public repository. For example, in addition to summary statistics, the data points behind means, medians and variance measures should be available. If there are restrictions on publicly sharing data—e.g. participant privacy or use of data from a third party—those must be specified.

Reviewer #1: No

Reviewer #2: Yes

4. Is the manuscript presented in an intelligible fashion and written in standard English?

PLOS ONE does not copyedit accepted manuscripts, so the language in submitted articles must be clear, correct, and unambiguous. Any typographical or grammatical errors should be corrected at revision, so please note any specific errors here.

Reviewer #1: Yes

Reviewer #2: Yes

5. Review Comments to the Author

Please use the space provided to explain your answers to the questions above. You may also include additional comments for the author, including concerns about dual publication, research ethics, or publication ethics. (Please upload your review as an attachment if it exceeds 20,000 characters)

Reviewer #1: 1) It would be appropriate to conclude the introduction with a brief overview of the overall structure of the article, not just the section immediately following. This would help the reader better navigate the argumentative and methodological path of the entire contribution.

**Response:**

We have closed the introduction with the following paragraph:

“The article is structured as follows. The next section outlines the theoretical framework underpinning the study, with particular attention to the relationship between universities and communication through platforms and the challenges of social media data classification. The Materials and Methods section then describes the case study design, data collection, and the classification procedures adopted by both human coders and AI models. The following section presents the Preliminary Analyses, which were conducted as a preparatory step toward the main findings. This section is followed by the Results section, which presents the outcomes of the comparison between human and automated classifications. The final section offers a concluding discussion of the findings, and their implications for future sociological research using AI tools.”.

A roadmap guiding the reader through the paper’s architecture is now included at the end of the introduction.

2) The section entitled Theoretical Framework is useful and well constructed, but it is suggested that a second review paragraph. It would be useful to recall studies that have compared classifications generated by artificial intelligence models with human classifications, or more generally work that has questioned the comparability between outputs produced by AI and those generated by human experts.

**Response:**

Thank you, we have added a review paragraph to the Theoretical framework, which discusses how LLM-based classification can be embedded into a researcher's workflow, in the absence of specific literature comparing AI-generated classification with those made by human experts.

3) The methodology section lacks the tool used to collect Facebook posts.

**Response:**

Thank you, we added the following sentence in the methodology section: “The text of the posts was extracted directly from the Facebook pages of the universities by parsing the page content saved through the browser.”

4) It would be helpful to explain why davinci-002 was specifically chosen. A brief note on any cost, time, or accessibility constraints would help clarify this methodological decision.

**Response:**

Thank you for the suggestion; we have added the following text:
"The second component involves introducing a zero-shot classification model, specifically the davinci-002 model accessed via the OpenAI API. This choice was motivated by the model’s high level of accuracy in text understanding and classification tasks, as well as its favorable balance between performance and cost, making it particularly suitable for large-scale experimentation within a constrained research budget."

5) In the Materials and Methods section, some results are anticipated. It would be preferable to keep the description of data collection, data description and chosen analysis procedures separate from the empirical results, possibly breaking the results section into two subsections to improve readability.

**Response:**

Thank you — your suggestions helped us clarify the methodological aspects and distinguish the preliminary analyses from the main results.

To this end, the *Materials and Methods* section has been separated from a newly added section titled *Preliminary Analyses*. The former provides a detailed explanation of the methodological process, including the use of diagrams, while the latter describes the steps of the preliminary analyses outlined in the methodological workflow.

6) “While some categories show high levels of agreement, others reveal substantial differences in interpretation.” Attempts at reflection on this point would be helpful. My suggestion is to try to explain why some categories show higher levels of agreement than others. It might be found useful in strengthening the analysis.

**Response:**
Thank you, we have added a dedicated paragraph in Section Materials and Methods about semantic ambiguity.

7) You may want to add a brief definition of what a “zero-shot classification” is.

**Response:**

Thank you — we have added a brief definition of the method where zero-shot classification is first mentioned.

8) The technical description of the BERT model is relevant to the Materials and Methods section, but its current placement is problematic for fluency. This is because, as explained earlier, some results are also already anticipated in the same section. It makes for heavy reading. It is again suggested that the section be reorganized.

**Response:**

Thank you. We have reorganized the methodological section by separating the methodological aspects from the preliminary results. As suggested, we have also reduced the detailed description of the model, as it is not central to the main focus of our study.

Reviewer #2:

1. Streamline and Focus the Introduction

The introduction lacks clarity and structure. The flow moves from categorization concepts, to hypotheses, to contributions, to goals, then a case study, followed by research questions and new objectives. This creates confusion.

➤ Recommendation: Define a single, concise research objective early in the introduction and structure the rest of the section around that.

**Response:**
We have restructured the introduction to foreground the central research question:

“How reliable are the classifications generated by AI models when compared to those made by human experts? More specifically, is the agreement between AI-generated classifications and those produced by researchers comparable to the level of agreement observed among the researchers themselves?”

This research objective is now stated explicitly, and the remainder of the introduction articulates the empirical context, and methodological design in direct relation to this focus.

2. Avoid Overstretching the Paper's Goals

The paper attempts to do too much—methodological testing, theoretical contribution, practical case study—all at once. This weakens the focus.

➤ Recommendation: Narrow the scope and sharpen the research question to align with a clearly stated and achievable goal.

**Response:**

We have revised the introduction to clearly define a single, focused research objective. This has strengthened the internal coherence of the manuscript and clarified its contribution to the field.

3. Inconsistent Language Standards

There is a mix of British and American English (e.g., "categorisation" vs. "behavioral").
➤ Recommendation: Standardize spelling and terminology across the manuscript.

**Response:**

Thank you, we have ensured consistency in the use of language throughout the manuscript.

4. Repetitive Content in Table 4

Table 4 contains duplicated or redundant lines.

➤ Recommendation: Review for repetition and present only relevant, non-redundant data.

**Response:**

Thank you for pointing that out — it was a formatting error introduced by Word, and it has now been corrected.

5. Excessive Use of Raw Numbers

The manuscript presents too many tables filled with numbers, making it difficult to interpret results.
➤ Recommendation: Use graphs or visualizations (e.g., bar charts) to summarize and compare performance metrics more effectively.

**Response:**

We appreciate the reviewer’s suggestion. In response, we have tables 4 and 5 with bar chart visualizations that summarize and compare the key results more effectively. These figures provide a clearer overview of inter-rater agreement (Kappa scores) and model performance across different training sources. We decided to keep the other tables because we believe they are more informative than a graphical representation.

6. Overly Technical Descriptions in Methods

The methods section contains long and generic explanations of models like BERT and transformer architectures.
➤ Recommendation: Trim or move technical descriptions to the theoretical framework section. Focus the main section on what was done and why in this specific study.

**Response:**

Thanks for the suggestion—We removed the more descriptive section on the BERT models to make the text more streamlined.

7. Lack of Fine-Tuning Details

The fine-tuning process for the BERT models lacks key methodological details:

O What proportion of the dataset was used for training vs. testing?
o How was the dataset split to ensure no data leakage between training and evaluation?
o Which researcher’s annotations were used, and how was inter-rater disagreement handled?

**Response:**

We appreciate the questions, which provided a valuable opportunity to clarify and better articulate the methodological choices made in this study. We added the following details:

“To train and evaluate the classification models, the dataset was randomly divided using an 80/20 holdout strategy: 80% of the data was used for training, while the remaining 20% was set aside for testing. The split was performed using a fixed random seed to ensure reproducibility and to prevent any data leakage between training and evaluation phases. Each post was assigned exclusively to either the training or test set, ensuring that the model was evaluated only on unseen data.

The training data consisted of manual classifications performed independently by three researchers, each with expertise in communication studies. Separate models were trained using the full set of annotations from each individual researcher, as well as using combined datasets created by merging randomly sampled annotations from multiple researchers (e.g., 50/50 or 1/3 splits). This strategy allowed us to assess not only the effectiveness of individual annotation-based models, but also how combining different human perspectives impacted model performance and generalizability.

Inter-rater disagreement was not resolved through adjudication or consensus; instead, it was treated as an analytical variable, enabling us to compare the agreement between AI-generated classifications and human annotations, as well as the agreement levels among the human annotators themselves.”

8. Weak Validation Protocol

It is not clear whether proper validation procedures (e.g., cross-validation, holdout set) were followed to avoid overfitting.

➤ Recommendation: Clarify how model evaluation was separated from training data.

**Response:**

Thank you for your question. As we mentioned, the use of a clearly separated holdout set provided a robust basis for evaluating model performance and mitigating the risk of overfitting.

9. Basic Conclusion

The conclusion merely states that Fleiss' Kappa values show moderate-to-substantial agreement between BERT models and human coders. This is a basic, expected outcome and does not offer new insight.

➤ Recommendation: Provide a more critical interpretation of the results. What are the implications for future sociological research using AI tools? What are the limitations?

**Response:**

We have expanded our Conclusions section to include theoretical limitations, practical implications, and recommendations for future research.

These revisions would greatly improve clarity, methodological rigor, and the overall contribution of the paper.

We encourage the authors to consider refining their research questions, clarifying their theoretical framing, and implementing more rigorous evaluation protocols if they choose to revise and submit elsewhere.

6. PLOS authors have the option to publish the peer review history of their article ([what does this mean?](https://eur01.safelinks.protection.outlook.com/?url=https%3A%2F%2Ftrack.editorialmanager.com%2FCL0%2Fhttps%3A%252F%252Fjournals.plos.org%252Fplosone%252Fs%252Feditorial-and-peer-review-process%2523loc-peer-review-history%2F1%2F010f019723bc9035-292039d6-ee4c-4174-ad4b-9ee1d7cbcb77-000000%2FoG5qu2qg1aS8RG2jtgtd3JdCksLkbIhJdPxOrJKE3Ss%3D213&data=05%7C02%7Canucita%40unime.it%7C3b041f7ee59144b863f408dd9fd9ee3b%7C84679d4583464e238c84a7304edba77f%7C0%7C0%7C638842480836554977%7CUnknown%7CTWFpbGZsb3d8eyJFbXB0eU1hcGkiOnRydWUsIlYiOiIwLjAuMDAwMCIsIlAiOiJXaW4zMiIsIkFOIjoiTWFpbCIsIldUIjoyfQ%3D%3D%7C0%7C%7C%7C&sdata=swSjYzms8idRCJoNW38uwPLlXKCTsz8AdlGjkAZwMWU%3D&reserved=0)). If published, this will include your full peer review and any attached files.
