# Supplementary material for: AI and social science: Automatic classification tools for big data analysis in sociological research
Source: PLoS One. 2026 Jun 18;21(6):e0351336. doi: 10.1371/journal.pone.0351336 (PMC13278443; doi:10.1371/journal.pone.0351336)
Supplement: Attachment — Submitted filename: Response to reviewers round 2.docx [file pone.0351336.s003.docx]

Additional Editor Comments:

After reviewing the revised manuscript, the reviewers indicate that some additional revisions are still needed. These are more limited in scope compared to the previous round, but necessary to further strengthen the paper. I kindly invite you to address them and submit a revised version.


[Note: HTML markup is below. Please do not edit.]

Reviewers' comments:

Reviewer's Responses to Questions

**Comments to the Author**

1. If the authors have adequately addressed your comments raised in a previous round of review and you feel that this manuscript is now acceptable for publication, you may indicate that here to bypass the “Comments to the Author” section, enter your conflict of interest statement in the “Confidential to Editor” section, and submit your "Accept" recommendation.

Reviewer #1: All comments have been addressed

Reviewer #3: All comments have been addressed

2. Is the manuscript technically sound, and do the data support the conclusions?

The manuscript must describe a technically sound piece of scientific research with data that supports the conclusions. Experiments must have been conducted rigorously, with appropriate controls, replication, and sample sizes. The conclusions must be drawn appropriately based on the data presented.

Reviewer #1: Yes

Reviewer #3: Partly

3. Has the statistical analysis been performed appropriately and rigorously?

Reviewer #1: Yes

Reviewer #3: I Don't Know

4. Have the authors made all data underlying the findings in their manuscript fully available?

The [PLOS Data policy](https://eur01.safelinks.protection.outlook.com/?url=http%3A%2F%2Ftrack.editorialmanager.com%2FCL0%2Fhttp%3A%252F%252Fwww.plosone.org%252Fstatic%252Fpolicies.action%2523sharing%2F1%2F010f019b4d99ad92-eb94e617-fb17-4977-a11a-825bb5192932-000000%2Fpw8-etJDJPl31xWnoHBuSJf-cXYIV2OzX-z16qdcGgs%3D242&data=05%7C02%7Canucita%40unime.it%7Cbfb88d8faee44ff4943508de4c4f4376%7C84679d4583464e238c84a7304edba77f%7C0%7C0%7C639032100475279000%7CUnknown%7CTWFpbGZsb3d8eyJFbXB0eU1hcGkiOnRydWUsIlYiOiIwLjAuMDAwMCIsIlAiOiJXaW4zMiIsIkFOIjoiTWFpbCIsIldUIjoyfQ%3D%3D%7C0%7C%7C%7C&sdata=m8nRq7ia3cNOpAYhhhjOnjLBbNQqK6KtV7ZLWvimJV4%3D&reserved=0) requires authors to make all data underlying the findings described in their manuscript fully available without restriction, with rare exception (please refer to the Data Availability Statement in the manuscript PDF file). The data should be provided as part of the manuscript or its supporting information, or deposited to a public repository. For example, in addition to summary statistics, the data points behind means, medians and variance measures should be available. If there are restrictions on publicly sharing data—e.g. participant privacy or use of data from a third party—those must be specified.

Reviewer #1: No

Reviewer #3: No

5. Is the manuscript presented in an intelligible fashion and written in standard English?

PLOS ONE does not copyedit accepted manuscripts, so the language in submitted articles must be clear, correct, and unambiguous. Any typographical or grammatical errors should be corrected at revision, so please note any specific errors here.

Reviewer #1: Yes

Reviewer #3: No

6. Review Comments to the Author

Please use the space provided to explain your answers to the questions above. You may also include additional comments for the author, including concerns about dual publication, research ethics, or publication ethics. (Please upload your review as an attachment if it exceeds 20,000 characters)

Reviewer #1: The authors have made a substantial effort in revising the manuscript. All the comments I previously raised have been addressed, and the manuscript is now much clearer, better structured, and more coherent.
There are, however, two aspects that would still benefit from further refinement.
First, the descriptive section on the BERT model remains relatively long and overly technical. I suggest focusing more specifically on what is necessary for understanding the study, as this would improve readability.

Response: Thank you for the suggestion; we have further reduced the section on BERT models, focusing instead on their ability to be adapted to a specific context through fine-tuning on labeled data. This aspect is particularly relevant in our context, as researchers can manually label a subset of the data, thereby enabling the model to leverage this additional domain knowledge to classify large volumes of text. The revised text is as follows:

“To enhance the automatic classification and create a model specifically trained with the researchers' classification data, we decided to apply a Bidirectional Encoder Representations from Transformers (BERT) model pre-trained in Italian (More information about the model is available here: https://huggingface.co/dbmdz/bert-base-italian-cased). This model was fine-tuned using the researchers' classification results. BERT (Bidirectional Encoder Representations from Transformers) models are particularly well-suited for text classification tasks for several reasons. Since their introduction in 2018 (Devlin et al., 2019), and have been proved to be among the most performant Large Language Models (LLMs) in the literature. BERT models can be fine-tuned on task-specific data, allowing them to be adapted to a particular analytical context; this is especially useful in our study, where the model is trained on classifications provided by researchers to align its predictions with domain-specific interpretative criteria. This approach not only enhances the reliability of automatic text classification but also reduces the need for extensive manual classification efforts, therefore aligning well with our research objectives.”

Second, although the Conclusions section is now more articulated, it could be strengthened by expanding the epistemological reflection on what it means for AI models to learn from divergent human classifications. Additionally, the brief reference to cybersecurity appears somewhat disconnected from the rest of the article; clarifying its relevance or removing it would enhance the coherence of the final section.

Response:
Thank you for this question, which allows us to clarify how these models can be situated within the context of text classification. We have added the following text:

“From a methodological and epistemological perspective, our analysis is grounded in the distinction between two complementary evaluation dimensions.
The first concerns model accuracy, which is primarily related to the fine-tuning process and can be interpreted as a technical performance measure, reflecting how effectively the model learns from labeled data.

The second dimension concerns the degree of agreement, both among human researchers and between researchers and AI models. In this case, misalignment should not be interpreted as a failure of the model, but rather as an informative result.

The introduction of an AI model is not intended to impose an objective or definitive interpretive framework—something that is neither feasible nor desirable in the context of semantically ambiguous sociological data. Instead, the goal is for the model to reproduce a level of variability comparable to that observed among human coders. In this sense, disagreement is not an error to be eliminated, but a characteristic of the classification task itself.

An AI model can therefore be considered suitable for large-scale data processing insofar as it does not diverge from human classifications in a substantially different way than researchers already diverge from one another. Under this perspective, fine-tuned models do not replace human interpretation, but align with it, functioning as scalable extensions of human classificatory practices rather than as arbiters of a single “correct” classification.”

The reference to cybersecurity is indeed of interest to us, particularly given the recent advances in AI-based classification systems for anomaly detection. However, we acknowledge that addressing this topic in a sufficiently rigorous and comprehensible manner would risk diverting attention from the main focus of the paper. For this reason, we have decided to remove the reference to cybersecurity in order to improve the coherence and clarity of the section.

Overall, the manuscript has improved significantly.

Reviewer #3: Dear authors,
To have your paper published, further work is needed:
1. The study significance, research gap, research design, research questions are not there in the abstract and the Key words are missing.

Response: Thank you for this comment. The abstract has been reformulated to explicitly address the study significance, research gap, research design, and research questions, and

keywords have been added accordingly.

2. The introduction section needs to be reconsidered in the sense of forgrounding the study significance, specifying the gap and ending with the research question. The last part of it about structing the article is out od lace, please remove it.

Response: Thank you for this comment. The Introduction has been partially revised, as highlighted in the manuscript, in order to better foreground the study significance, specify the research gap, and clearly state the research questions. In addition, the final part describing the structure of the article has been removed, as requested.


3. The APA system of writing is not consistently used throughout the paper.

Response: Thank you for this comment. We have revised and standardized the references to ensure consistent use of the citation style throughout the paper.

4. The title 'Theoretical Framework' should be replaced with 'Literature Review' and should be reconsidered to have subsections.

Response:

Thank you for the suggestion. We have renamed the section “Literature Review” and reorganized it into the following subsections:

- Social networks as platforms
- Social media in public communication
- Public institutional communication
- Manual and automated post classification

5. There are many language problems such as the sentence immediately before Table 2.

Response: Thank you, we have carefully reviewed the English and corrected some typos.

6. I is preferable to have 'Analysis' instead of 'Preliminary analyses' where steps 1 ad 2 are replaced with 'Research question1' and 'Research question 2' to to better correlate the questions to the analysis section.

Response: Thank you. We have modified the section titles as suggested.

7. The discussion section should apear as 'Discussion' rather than concluding discussion.

Response: Thank you for the suggestion. We have expanded the discussion, also in response to the requests of Reviewer 1, and have therefore separated the Discussion from the Conclusions as suggested.

8. Having short paragraphs is disctructing specially one- sentence or two-sentence paragraphs. Somtimes ideas need have a page to be fully addressed.

Response: Thank you for this observation. While we recognize that some arguments may require extended discussion, the structure of the paper reflects common conventions in scientific writing, where clarity and conciseness are prioritized due to space limitations. Short paragraphs were used to emphasize specific points and to avoid conflating distinct aspects of the analysis.

9. The 'Conclusion' section is missing. And it is better to have a section titled 'Limitations and suggestions' where the authors identify their study limitations followed by suggestions for further future studies.

Response: Thank you for the suggestion. As mentioned above, we have separated the Discussion from the Conclusion. Regarding the limitations of the study, we chose to address them within the Discussion section, as we believe this approach supports a more cohesive interpretation of the results and helps readers better understand the scope and implications of each finding.

10. The list of references should be reconsidered to correctly use the system of writing followed in the study.

Response: Thank you for this comment. We have carefully revised the list of references and standardized it according to a consistent citation style used throughout the manuscript.

7. PLOS authors have the option to publish the peer review history of their article ([what does this mean?](https://eur01.safelinks.protection.outlook.com/?url=https%3A%2F%2Ftrack.editorialmanager.com%2FCL0%2Fhttps%3A%252F%252Fjournals.plos.org%252Fplosone%252Fs%252Feditorial-and-peer-review-process%2523loc-peer-review-history%2F1%2F010f019b4d99ad92-eb94e617-fb17-4977-a11a-825bb5192932-000000%2FeMEiO12LXAeCP2W9o9v6YQ5Ca_g5CVzvhDDh5rmCCbc%3D242&data=05%7C02%7Canucita%40unime.it%7Cbfb88d8faee44ff4943508de4c4f4376%7C84679d4583464e238c84a7304edba77f%7C0%7C0%7C639032100475296394%7CUnknown%7CTWFpbGZsb3d8eyJFbXB0eU1hcGkiOnRydWUsIlYiOiIwLjAuMDAwMCIsIlAiOiJXaW4zMiIsIkFOIjoiTWFpbCIsIldUIjoyfQ%3D%3D%7C0%7C%7C%7C&sdata=OZY7gdjQHr0Cw53%2FPWODqWjC868sO6zbrVWdICEBB0w%3D&reserved=0)). If published, this will include your full peer review and any attached files.

If you choose “no”, your identity will remain anonymous but your review may still be made public.


**Do you want your identity to be public for this peer review?** For information about this choice, including consent withdrawal, please see our [Privacy Policy](https://eur01.safelinks.protection.outlook.com/?url=https%3A%2F%2Ftrack.editorialmanager.com%2FCL0%2Fhttps%3A%252F%252Fwww.plos.org%252Fprivacy-policy%2F1%2F010f019b4d99ad92-eb94e617-fb17-4977-a11a-825bb5192932-000000%2F6Z-vXcm-AtDsmJOx5C_Vakugdq6xZvo06tiwbAuDagM%3D242&data=05%7C02%7Canucita%40unime.it%7Cbfb88d8faee44ff4943508de4c4f4376%7C84679d4583464e238c84a7304edba77f%7C0%7C0%7C639032100475313390%7CUnknown%7CTWFpbGZsb3d8eyJFbXB0eU1hcGkiOnRydWUsIlYiOiIwLjAuMDAwMCIsIlAiOiJXaW4zMiIsIkFOIjoiTWFpbCIsIldUIjoyfQ%3D%3D%7C0%7C%7C%7C&sdata=jH4AIzDEZaeaNGGx94bL7BKLnaqdmqU%2BSPHQapLYACI%3D&reserved=0).

Reviewer #1: No

Reviewer #3: **Yes:** Nawal Fadhil Abbas
