# Supplementary material for: AI and social science: Automatic classification tools for big data analysis in sociological research
Source: PLoS One. 2026 Jun 18;21(6):e0351336. doi: 10.1371/journal.pone.0351336 (PMC13278443; doi:10.1371/journal.pone.0351336)
Supplement: Attachment — Submitted filename: Response to reviewers round 3.docx [file pone.0351336.s004.docx]

Additional Editor Comments:

Dear Authors,

the reviewers find the manuscript improved, but some revisions are still required before it can be accepted for publication.

Kind regards,


[Note: HTML markup is below. Please do not edit.]

Reviewers' comments:

Reviewer's Responses to Questions

**Comments to the Author**

1. If the authors have adequately addressed your comments raised in a previous round of review and you feel that this manuscript is now acceptable for publication, you may indicate that here to bypass the “Comments to the Author” section, enter your conflict of interest statement in the “Confidential to Editor” section, and submit your "Accept" recommendation.

Reviewer #1: All comments have been addressed

Reviewer #4: All comments have been addressed

2. Is the manuscript technically sound, and do the data support the conclusions?

The manuscript must describe a technically sound piece of scientific research with data that supports the conclusions. Experiments must have been conducted rigorously, with appropriate controls, replication, and sample sizes. The conclusions must be drawn appropriately based on the data presented.

Reviewer #1: Yes

Reviewer #4: Yes

3. Has the statistical analysis been performed appropriately and rigorously?

Reviewer #1: Yes

Reviewer #4: No

4. Have the authors made all data underlying the findings in their manuscript fully available?

The [PLOS Data policy](https://eur01.safelinks.protection.outlook.com/?url=http%3A%2F%2Ftrack.editorialmanager.com%2FCL0%2Fhttp%3A%252F%252Fwww.plosone.org%252Fstatic%252Fpolicies.action%2523sharing%2F1%2F010f019d6fd0095d-8c21c8d0-06de-497d-bccd-28d4dbf2a4c6-000000%2FIJVqRvx22fu9PzCj0JVIsgXAtpZePBVXvMoR3-63RE8%3D258&data=05%7C02%7Canucita%40unime.it%7Cc413e246431d4c88432408de9607d5e6%7C84679d4583464e238c84a7304edba77f%7C0%7C0%7C639113157550212009%7CUnknown%7CTWFpbGZsb3d8eyJFbXB0eU1hcGkiOnRydWUsIlYiOiIwLjAuMDAwMCIsIlAiOiJXaW4zMiIsIkFOIjoiTWFpbCIsIldUIjoyfQ%3D%3D%7C0%7C%7C%7C&sdata=t1JIoYkYM7Ws%2FXbRFcrdwunVjEvZhLXLe%2F7jbCb52kg%3D&reserved=0) requires authors to make all data underlying the findings described in their manuscript fully available without restriction, with rare exception (please refer to the Data Availability Statement in the manuscript PDF file). The data should be provided as part of the manuscript or its supporting information, or deposited to a public repository. For example, in addition to summary statistics, the data points behind means, medians and variance measures should be available. If there are restrictions on publicly sharing data—e.g. participant privacy or use of data from a third party—those must be specified.

Reviewer #1: Yes

Reviewer #4: No

5. Is the manuscript presented in an intelligible fashion and written in standard English?

PLOS ONE does not copyedit accepted manuscripts, so the language in submitted articles must be clear, correct, and unambiguous. Any typographical or grammatical errors should be corrected at revision, so please note any specific errors here.

Reviewer #1: Yes

Reviewer #4: No

6. Review Comments to the Author

Please use the space provided to explain your answers to the questions above. You may also include additional comments for the author, including concerns about dual publication, research ethics, or publication ethics. (Please upload your review as an attachment if it exceeds 20,000 characters)

Reviewer #1: In my opinion, all the comments raised in the previous rounds of review have been satisfactorily addressed.

Reviewer #4: The manuscript has clearly improved in structure, clarity, and overall coherence compared to the previous version. However, some aspects still require further refinement before publication. In particular, the data availability statement should be strengthened to clarify more explicitly what data are accessible and to what extent the analysis can be replicated, in line with journal requirements.

**Response**: Thank you for your comment. We have updated the Data Availability statement to more clearly specify which data are publicly available and to explicitly address the replicability of the analysis.

*Revised Data Availability Statement*

The raw data (i.e., Facebook posts from the official pages of the University of Messina and the University of Salerno) cannot be publicly shared due to Meta’s data usage policies, which prohibit the redistribution of content extracted from the platform.

However, the annotated datasets and trained models used for AI model development are publicly available on our Hugging Face repository:
<https://huggingface.co/HuM-HILab/pub_comm_classification>

These resources include the full annotation schema, data structure, and model configurations used in the study, and are intended to enable transparency and reproducibility of the analytical workflow. While the original post texts are not included, the shared materials provide all necessary components to replicate the methodology.

Researchers who have independent access to comparable raw data (i.e., post texts) can reproduce the analysis using the provided annotations and models, or apply the same pipeline to alternative datasets. For further details on the data collection and processing procedures, researchers may contact the HuM-HI Laboratory at humhilab@unime.it or visit humhilab.unime.it.

The description of the statistical approach would also benefit from greater transparency, especially regarding the measures of agreement used and their interpretation.

**Response:** Thank you for your comment. We have expanded the methodological section to clarify the rationale for selecting Fleiss’ Kappa, highlighting its appropriateness for multi-rater agreement and its distinction from Cohen’s Kappa.

Additionally, the manuscript still presents some language issues, with sentences that are occasionally overly complex or not fully fluent, and would benefit from careful editing.

**Response:** Thank you for your comment. We have carefully revised the entire manuscript, paying particular attention to improving clarity and fluency, and simplifying sentences that were overly complex or unclear.

Finally, the introduction and the section on BERT could be further streamlined to improve focus and readability,

**Response:** Thank you for your comment. We have made revisions where possible to improve the clarity and flow of both the Introduction and the section on BERT, while retaining the key references and information needed for a proper understanding of the research.

and the study's limitations should be articulated more clearly, ideally in a more visible, structured way.

**Response:** Thank you for your comment. We have revised this section to more clearly articulate the study’s limitations, organizing them in a dedicated and structured way. We have also clarified the role of the empirical context, emphasizing that while it may limit generalizability, the primary contribution of the study is methodological.

7. PLOS authors have the option to publish the peer review history of their article ([what does this mean?](https://eur01.safelinks.protection.outlook.com/?url=https%3A%2F%2Ftrack.editorialmanager.com%2FCL0%2Fhttps%3A%252F%252Fjournals.plos.org%252Fplosone%252Fs%252Feditorial-and-peer-review-process%2523loc-peer-review-history%2F1%2F010f019d6fd0095d-8c21c8d0-06de-497d-bccd-28d4dbf2a4c6-000000%2FJg0FOdC3SPkMH-UDGfC4wBQyMiB82gGQ_PnwfGp3Jlc%3D258&data=05%7C02%7Canucita%40unime.it%7Cc413e246431d4c88432408de9607d5e6%7C84679d4583464e238c84a7304edba77f%7C0%7C0%7C639113157550259026%7CUnknown%7CTWFpbGZsb3d8eyJFbXB0eU1hcGkiOnRydWUsIlYiOiIwLjAuMDAwMCIsIlAiOiJXaW4zMiIsIkFOIjoiTWFpbCIsIldUIjoyfQ%3D%3D%7C0%7C%7C%7C&sdata=k36K5RGVlPFLnKd5tvGvaSsoD6PwlC1cM3tdXSDJEcQ%3D&reserved=0)). If published, this will include your full peer review and any attached files.


If you choose “no”, your identity will remain anonymous but your review may still be made public.


**Do you want your identity to be public for this peer review?** For information about this choice, including consent withdrawal, please see our [Privacy Policy](https://eur01.safelinks.protection.outlook.com/?url=https%3A%2F%2Ftrack.editorialmanager.com%2FCL0%2Fhttps%3A%252F%252Fwww.plos.org%252Fprivacy-policy%2F1%2F010f019d6fd0095d-8c21c8d0-06de-497d-bccd-28d4dbf2a4c6-000000%2FyirA9_QOUlYM9TSljntIHP58iDHYTGLfo4WrecfxUcM%3D258&data=05%7C02%7Canucita%40unime.it%7Cc413e246431d4c88432408de9607d5e6%7C84679d4583464e238c84a7304edba77f%7C0%7C0%7C639113157550309861%7CUnknown%7CTWFpbGZsb3d8eyJFbXB0eU1hcGkiOnRydWUsIlYiOiIwLjAuMDAwMCIsIlAiOiJXaW4zMiIsIkFOIjoiTWFpbCIsIldUIjoyfQ%3D%3D%7C0%7C%7C%7C&sdata=QdEk79Sg9Gk8xHDojvgetChkdqNoQcE6QozO8ocRaHk%3D&reserved=0).

Reviewer #1: No

Reviewer #4: No
