# Supplementary material for: AI and social science: Automatic classification tools for big data analysis in sociological research
Source: PLoS One. 2026 Jun 18;21(6):e0351336. doi: 10.1371/journal.pone.0351336 (PMC13278443; doi:10.1371/journal.pone.0351336)
Supplement: Peer review history

# Peer review: Decision Letter 0

**DOI:** 10.1371/journal.pone.0351336.r001

**Authors:** Ciro Clemente De Falco

30 May 2025

PONE-D-25-21922

AI and Social Science. Automatic Classification Tools for Big Data Analysis in Sociological Research

PLOS ONE

Dear Dr. Nucita,

Thank you for submitting your manuscript to PLOS ONE. After careful consideration, we feel that it has merit but does not fully meet PLOS ONE’s publication criteria as it currently stands. Therefore, we invite you to submit a revised version of the manuscript that addresses the points raised during the review process.

Please submit your revised manuscript by Jul 13 2025 11:59PM. If you will need more time than this to complete your revisions, please reply to this message or contact the journal office at plosone@plos.org. When you're ready to submit your revision, log on to https://www.editorialmanager.com/pone/ and select the 'Submissions Needing Revision' folder to locate your manuscript file.

Please include the following items when submitting your revised manuscript:

- A rebuttal letter that responds to each point raised by the academic editor and reviewer(s). You should upload this letter as a separate file labeled 'Response to Reviewers'.
- A marked-up copy of your manuscript that highlights changes made to the original version. You should upload this as a separate file labeled 'Revised Manuscript with Track Changes'.
- An unmarked version of your revised paper without tracked changes. You should upload this as a separate file labeled 'Manuscript'.

If you would like to make changes to your financial disclosure, please include your updated statement in your cover letter. Guidelines for resubmitting your figure files are available below the reviewer comments at the end of this letter.

If applicable, we recommend that you deposit your laboratory protocols in protocols.io to enhance the reproducibility of your results. Protocols.io assigns your protocol its own identifier (DOI) so that it can be cited independently in the future. For instructions see: https://journals.plos.org/plosone/s/submission-guidelines#loc-laboratory-protocols. Additionally, PLOS ONE offers an option for publishing peer-reviewed Lab Protocol articles, which describe protocols hosted on protocols.io. Read more information on sharing protocols at https://plos.org/protocols?utm_medium=editorial-email&utm_source=authorletters&utm_campaign=protocols.

We look forward to receiving your revised manuscript.

Kind regards,

Ciro Clemente De Falco

Academic Editor

PLOS ONE

Journal Requirements:

When submitting your revision, we need you to address these additional requirements.

1. Please ensure that your manuscript meets PLOS ONE's style requirements, including those for file naming. The PLOS ONE style templates can be found at https://journals.plos.org/plosone/s/file?id=wjVg/PLOSOne_formatting_sample_main_body.pdf and https://journals.plos.org/plosone/s/file?id=ba62/PLOSOne_formatting_sample_title_authors_affiliations.pdf

2. In your Methods section, please include additional information about your dataset and ensure that you have included a statement specifying whether the collection and analysis method complied with the terms and conditions for the source of the data.

3. Please note that PLOS ONE has specific guidelines on code sharing for submissions in which author-generated code underpins the findings in the manuscript. In these cases, we expect all author-generated code to be made available without restrictions upon publication of the work. Please review our guidelines at https://journals.plos.org/plosone/s/materials-and-software-sharing#loc-sharing-code and ensure that your code is shared in a way that follows best practice and facilitates reproducibility and reuse.

4. We note that you have indicated that there are restrictions to data sharing for this study. PLOS only allows data to be available upon request if there are legal or ethical restrictions on sharing data publicly. For more information on unacceptable data access restrictions, please see http://journals.plos.org/plosone/s/data-availability#loc-unacceptable-data-access-restrictions.

Before we proceed with your manuscript, please address the following prompts:

a) If there are ethical or legal restrictions on sharing a de-identified data set, please explain them in detail (e.g., data contain potentially identifying or sensitive patient information, data are owned by a third-party organization, etc.) and who has imposed them (e.g., a Research Ethics Committee or Institutional Review Board, etc.). Please also provide contact information for a data access committee, ethics committee, or other institutional body to which data requests may be sent.

b) If there are no restrictions, please upload the minimal anonymized data set necessary to replicate your study findings to a stable, public repository and provide us with the relevant URLs, DOIs, or accession numbers. For a list of recommended repositories, please see

https://journals.plos.org/plosone/s/recommended-repositories. You also have the option of uploading the data as Supporting Information files, but we would recommend depositing data directly to a data repository if possible.

We will update your Data Availability statement on your behalf to reflect the information you provide.

5. Please provide a complete Data Availability Statement in the submission form, ensuring you include all necessary access information or a reason for why you are unable to make your data freely accessible. If your research concerns only data provided within your submission, please write "All data are in the manuscript and/or supporting information files" as your Data Availability Statement.

6. We note you have included a table to which you do not refer in the text of your manuscript. Please ensure that you refer to Table 2, 3, 4, in your text; if accepted, production will need this reference to link the reader to the Table.

Additional Editor Comments :

Dear Author,

We have now received the reports from two reviewers.

The evaluations differ: while one reviewer recommends rejection, the other suggests only minor revisions. After carefully considering both reports and conducting an overall assessment of your submission, I have decided to invite you to submit a major revision.

Although there are notable limitations in the current version both reviewers agree that the manuscript addresses a promising and relevant topic. I believe that with substantial revisions, the article has the potential to make a valuable contribution.

Please respond thoroughly to each of the reviewers’ comments and revise your manuscript accordingly. I encourage you to use this opportunity to strengthen the conceptual framing and overall coherence of the article.

Should you have any questions, feel free to get in touch.

Best regards,

[Note: HTML markup is below. Please do not edit.]

Reviewers' comments:

Reviewer's Responses to Questions

**Comments to the Author**

1. Is the manuscript technically sound, and do the data support the conclusions?

The manuscript must describe a technically sound piece of scientific research with data that supports the conclusions. Experiments must have been conducted rigorously, with appropriate controls, replication, and sample sizes. The conclusions must be drawn appropriately based on the data presented.

Reviewer #1: Yes

Reviewer #2: Partly

**********

2. Has the statistical analysis been performed appropriately and rigorously?

Reviewer #1: Yes

Reviewer #2: Yes

**********

3. Have the authors made all data underlying the findings in their manuscript fully available?

The PLOS Data policy requires authors to make all data underlying the findings described in their manuscript fully available without restriction, with rare exception (please refer to the Data Availability Statement in the manuscript PDF file). The data should be provided as part of the manuscript or its supporting information, or deposited to a public repository. For example, in addition to summary statistics, the data points behind means, medians and variance measures should be available. If there are restrictions on publicly sharing data—e.g. participant privacy or use of data from a third party—those must be specified.

Reviewer #1: No

Reviewer #2: Yes

**********

4. Is the manuscript presented in an intelligible fashion and written in standard English?

PLOS ONE does not copyedit accepted manuscripts, so the language in submitted articles must be clear, correct, and unambiguous. Any typographical or grammatical errors should be corrected at revision, so please note any specific errors here.

Reviewer #1: Yes

Reviewer #2: Yes

**********

5. Review Comments to the Author

Please use the space provided to explain your answers to the questions above. You may also include additional comments for the author, including concerns about dual publication, research ethics, or publication ethics. (Please upload your review as an attachment if it exceeds 20,000 characters)

Reviewer #1: 1) It would be appropriate to conclude the introduction with a brief overview of the overall structure of the article, not just the section immediately following. This would help the reader better navigate the argumentative and methodological path of the entire contribution.

2) The section entitled Theoretical Framework is useful and well constructed, but it is suggested that a second review paragraph. It would be useful to recall studies that have compared classifications generated by artificial intelligence models with human classifications, or more generally work that has questioned the comparability between outputs produced by AI and those generated by human experts.

3) The methodology section lacks the tool used to collect Facebook posts.

4) It would be helpful to explain why davinci-002 was specifically chosen. A brief note on any cost, time, or accessibility constraints would help clarify this methodological decision.

5) In the Materials and Methods section, some results are anticipated. It would be preferable to keep the description of data collection, data description and chosen analysis procedures separate from the empirical results, possibly breaking the results section into two subsections to improve readability.

6) “While some categories show high levels of agreement, others reveal substantial differences in interpretation.” Attempts at reflection on this point would be helpful. My suggestion is to try to explain why some categories show higher levels of agreement than others. It might be found useful in strengthening the analysis.

7) You may want to add a brief definition of what a “zero-shot classification” is.

8) The technical description of the BERT model is relevant to the Materials and Methods section, but its current placement is problematic for fluency. This is because, as explained earlier, some results are also already anticipated in the same section. It makes for heavy reading. It is again suggested that the section be reorganized.

Reviewer #2: 1. Streamline and Focus the Introduction

The introduction lacks clarity and structure. The flow moves from categorization concepts, to hypotheses, to contributions, to goals, then a case study, followed by research questions and new objectives. This creates confusion.

➤ Recommendation: Define a single, concise research objective early in the introduction and structure the rest of the section around that.

2. Avoid Overstretching the Paper's Goals

The paper attempts to do too much—methodological testing, theoretical contribution, practical case study—all at once. This weakens the focus.

➤ Recommendation: Narrow the scope and sharpen the research question to align with a clearly stated and achievable goal.

3. Inconsistent Language Standards

There is a mix of British and American English (e.g., "categorisation" vs. "behavioral").

➤ Recommendation: Standardize spelling and terminology across the manuscript.

4. Repetitive Content in Table 4

Table 4 contains duplicated or redundant lines.

➤ Recommendation: Review for repetition and present only relevant, non-redundant data.

5. Excessive Use of Raw Numbers

The manuscript presents too many tables filled with numbers, making it difficult to interpret results.

➤ Recommendation: Use graphs or visualizations (e.g., bar charts) to summarize and compare performance metrics more effectively.

6. Overly Technical Descriptions in Methods

The methods section contains long and generic explanations of models like BERT and transformer architectures.

➤ Recommendation: Trim or move technical descriptions to the theoretical framework section. Focus the main section on what was done and why in this specific study.

7. Lack of Fine-Tuning Details

The fine-tuning process for the BERT models lacks key methodological details:

o What proportion of the dataset was used for training vs. testing?

o How was the dataset split to ensure no data leakage between training and evaluation?

o Which researcher’s annotations were used, and how was inter-rater disagreement handled?

8. Weak Validation Protocol

It is not clear whether proper validation procedures (e.g., cross-validation, holdout set) were followed to avoid overfitting.

➤ Recommendation: Clarify how model evaluation was separated from training data.

9. Basic Conclusion

The conclusion merely states that Fleiss' Kappa values show moderate-to-substantial agreement between BERT models and human coders. This is a basic, expected outcome and does not offer new insight.

➤ Recommendation: Provide a more critical interpretation of the results. What are the implications for future sociological research using AI tools? What are the limitations?

These revisions would greatly improve clarity, methodological rigor, and the overall contribution of the paper.

We encourage the authors to consider refining their research questions, clarifying their theoretical framing, and implementing more rigorous evaluation protocols if they choose to revise and submit elsewhere.

**********

6. PLOS authors have the option to publish the peer review history of their article (what does this mean?). If published, this will include your full peer review and any attached files.

If you choose “no”, your identity will remain anonymous but your review may still be made public.

**Do you want your identity to be public for this peer review?**   For information about this choice, including consent withdrawal, please see our Privacy Policy.

Reviewer #1: No

Reviewer #2: **Yes:**  Wesley Lourenco Barbosa

**********

[NOTE: If reviewer comments were submitted as an attachment file, they will be attached to this email and accessible via the submission site. Please log into your account, locate the manuscript record, and check for the action link "View Attachments". If this link does not appear, there are no attachment files.]

While revising your submission, please upload your figure files to the Preflight Analysis and Conversion Engine (PACE) digital diagnostic tool, https://pacev2.apexcovantage.com/. PACE helps ensure that figures meet PLOS requirements. To use PACE, you must first register as a user. Registration is free. Then, login and navigate to the UPLOAD tab, where you will find detailed instructions on how to use the tool. If you encounter any issues or have any questions when using PACE, please email PLOS at figures@plos.org. Please note that Supporting Information files do not need this step.

# Author comment: Author response to Decision Letter 1

**DOI:** 10.1371/journal.pone.0351336.r002

24 Nov 2025

Reviewer #1: 1) It would be appropriate to conclude the introduction with a brief overview of the overall structure of the article, not just the section immediately following. This would help the reader better navigate the argumentative and methodological path of the entire contribution.

Response:

We have closed the introduction with the following paragraph:

“The article is structured as follows. The next section outlines the theoretical framework underpinning the study, with particular attention to the relationship between universities and communication through platforms and the challenges of social media data classification. The Materials and Methods section then describes the case study design, data collection, and the classification procedures adopted by both human coders and AI models. The following section presents the Preliminary Analyses, which were conducted as a preparatory step toward the main findings. This section is followed by the Results section, which presents the outcomes of the comparison between human and automated classifications. The final section offers a concluding discussion of the findings, and their implications for future sociological research using AI tools.”.

A roadmap guiding the reader through the paper’s architecture is now included at the end of the introduction.

2) The section entitled Theoretical Framework is useful and well constructed, but it is suggested that a second review paragraph. It would be useful to recall studies that have compared classifications generated by artificial intelligence models with human classifications, or more generally work that has questioned the comparability between outputs produced by AI and those generated by human experts.

Response:

Thank you, we have added a review paragraph to the Theoretical framework, which discusses how LLM-based classification can be embedded into a researcher's workflow, in the absence of specific literature comparing AI-generated classification with those made by human experts.

3) The methodology section lacks the tool used to collect Facebook posts.

Response:

Thank you, we added the following sentence in the methodology section: “The text of the posts was extracted directly from the Facebook pages of the universities by parsing the page content saved through the browser.”

4) It would be helpful to explain why davinci-002 was specifically chosen. A brief note on any cost, time, or accessibility constraints would help clarify this methodological decision.

Response:

Thank you for the suggestion; we have added the following text:

"The second component involves introducing a zero-shot classification model, specifically the davinci-002 model accessed via the OpenAI API. This choice was motivated by the model’s high level of accuracy in text understanding and classification tasks, as well as its favorable balance between performance and cost, making it particularly suitable for large-scale experimentation within a constrained research budget."

5) In the Materials and Methods section, some results are anticipated. It would be preferable to keep the description of data collection, data description and chosen analysis procedures separate from the empirical results, possibly breaking the results section into two subsections to improve readability.

Response:

Thank you — your suggestions helped us clarify the methodological aspects and distinguish the preliminary analyses from the main results.

To this end, the Materials and Methods section has been separated from a newly added section titled Preliminary Analyses. The former provides a detailed explanation of the methodological process, including the use of diagrams, while the latter describes the steps of the preliminary analyses outlined in the methodological workflow.

6) “While some categories show high levels of agreement, others reveal substantial differences in interpretation.” Attempts at reflection on this point would be helpful. My suggestion is to try to explain why some categories show higher levels of agreement than others. It might be found useful in strengthening the analysis.

Response:

Thank you, we have added a dedicated paragraph in Section Materials and Methods about semantic ambiguity.

7) You may want to add a brief definition of what a “zero-shot classification” is.

Response:

Thank you — we have added a brief definition of the method where zero-shot classification is first mentioned.

8) The technical description of the BERT model is relevant to the Materials and Methods section, but its current placement is problematic for fluency. This is because, as explained earlier, some results are also already anticipated in the same section. It makes for heavy reading. It is again suggested that the section be reorganized.

Response:

Thank you. We have reorganized the methodological section by separating the methodological aspects from the preliminary results. As suggested, we have also reduced the detailed description of the model, as it is not central to the main focus of our study.

Reviewer #2:

1. Streamline and Focus the Introduction

The introduction lacks clarity and structure. The flow moves from categorization concepts, to hypotheses, to contributions, to goals, then a case study, followed by research questions and new objectives. This creates confusion.

➤ Recommendation: Define a single, concise research objective early in the introduction and structure the rest of the section around that.

Response:

We have restructured the introduction to foreground the central research question:

“How reliable are the classifications generated by AI models when compared to those made by human experts? More specifically, is the agreement between AI-generated classifications and those produced by researchers comparable to the level of agreement observed among the researchers themselves?”

This research objective is now stated explicitly, and the remainder of the introduction articulates the empirical context, and methodological design in direct relation to this focus.

2. Avoid Overstretching the Paper's Goals

The paper attempts to do too much—methodological testing, theoretical contribution, practical case study—all at once. This weakens the focus.

➤ Recommendation: Narrow the scope and sharpen the research question to align with a clearly stated and achievable goal.

Response:

We have revised the introduction to clearly define a single, focused research objective. This has strengthened the internal coherence of the manuscript and clarified its contribution to the field.

3. Inconsistent Language Standards

There is a mix of British and American English (e.g., "categorisation" vs. "behavioral").

➤ Recommendation: Standardize spelling and terminology across the manuscript.

Response:

Thank you, we have ensured consistency in the use of language throughout the manuscript.

4. Repetitive Content in Table 4

Table 4 contains duplicated or redundant lines.

➤ Recommendation: Review for repetition and present only relevant, non-redundant data.

Response:

Thank you for pointing that out — it was a formatting error introduced by Word, and it has now been corrected.

5. Excessive Use of Raw Numbers

The manuscript presents too many tables filled with numbers, making it difficult to interpret results.

➤ Recommendation: Use graphs or visualizations (e.g., bar charts) to summarize and compare performance metrics more effectively.

Response:

We appreciate the reviewer’s suggestion. In response, we have tables 4 and 5 with bar chart visualizations that summarize and compare the key results more effectively. These figures provide a clearer overview of inter-rater agreement (Kappa scores) and model performance across different training sources. We decided to keep the other tables because we believe they are more informative than a graphical representation.

6. Overly Technical Descriptions in Methods

The methods section contains long and generic explanations of models like BERT and transformer architectures.

➤ Recommendation: Trim or move technical descriptions to the theoretical framework section. Focus the main section on what was done and why in this specific study.

Response:

Thanks for the suggestion—We removed the more descriptive section on the BERT models to make the text more streamlined.

7. Lack of Fine-Tuning Details

The fine-tuning process for the BERT models lacks key methodological details:

O What proportion of the dataset was used for training vs. testing?

o How was the dataset split to ensure no data leakage between training and evaluation?

o Which researcher’s annotations were used, and how was inter-rater disagreement handled?

Response:

We appreciate the questions, which provided a valuable opportunity to clarify and better articulate the methodological choices made in this study. We added the following details:

“To train and evaluate the classification models, the dataset was randomly divided using an 80/20 holdout strategy: 80% of the data was used for training, while the remaining 20% was set aside for testing. The split was performed using a fixed random seed to ensure reproducibility and to prevent any data leakage between training and evaluation phases. Each post was assigned exclusively to either the training or test set, ensuring that the model was evaluated only on unseen data.

The training data consisted of manual classifications performed independently by three researchers, each with expertise in communication studies. Separate models were trained using the full set of annotations from each individual researcher, as well as using combined datasets created by merging randomly sampled annotations from multiple researchers (e.g., 50/50 or 1/3 splits). This strategy allowed us to assess not only the effectiveness of individual annotation-based models, but also how combining different human perspectives impacted model performance and generalizability.

Inter-rater disagreement was not resolved through adjudication or consensus; instead, it was treated as an analytical variable, enabling us to compare the agreement between AI-generated classifications and human annotations, as well as the agreement levels among the human annotators themselves.”

8. Weak Validation Protocol

It is not clear whether proper validation procedures (e.g., cross-validation, holdout set) were followed to avoid overfitting.

➤ Recommendation: Clarify how model evaluation was separated from training data.

Response:

Thank you for your question. As we mentioned, the use of a clearly separated holdout set provided a robust basis for evaluating model performance and mitigating the risk of overfitting.

9. Basic Conclusion

The conclusion merely states that Fleiss' Kappa values show moderate-to-substantial agreement between BERT models and human coders. This is a basic, expected outcome and does not offer new insight.

➤ Recommendation: Provide a more critical interpretation of the results. What are the implications for future sociological research using AI tools? What are the limitations?

Response:

We have expanded our Conclusions section to include theoretical limitations, practical implications, and recommendations for future research.

These revisions would greatly improve clarity, methodological rigor, and the overall contribution of the paper.

We encourage the authors to consider refining their research questions, clarifying their theoretical framing, and implementing more rigorous evaluation protocols if they choose to revise and submit elsewhere.

Attachment Submitted filename: Response to reviewers041125.docx

# Peer review: Decision Letter 1

**DOI:** 10.1371/journal.pone.0351336.r003

**Authors:** Ciro Clemente De Falco

23 Dec 2025

PONE-D-25-21922R1

AI and Social Science. Automatic Classification Tools for Big Data Analysis in Sociological Research

PLOS One

Dear Dr. Nucita,

Thank you for submitting your manuscript to PLOS ONE. After careful consideration, we feel that it has merit but does not fully meet PLOS ONE’s publication criteria as it currently stands. Therefore, we invite you to submit a revised version of the manuscript that addresses the points raised during the review process.

Please submit your revised manuscript by Feb 06 2026 11:59PM. If you will need more time than this to complete your revisions, please reply to this message or contact the journal office at plosone@plos.org. When you're ready to submit your revision, log on to https://www.editorialmanager.com/pone/ and select the 'Submissions Needing Revision' folder to locate your manuscript file.

Please include the following items when submitting your revised manuscript:

- A letter that responds to each point raised by the academic editor and reviewer(s). You should upload this letter as a separate file labeled 'Response to Reviewers'.
- A marked-up copy of your manuscript that highlights changes made to the original version. You should upload this as a separate file labeled 'Revised Manuscript with Track Changes'.
- An unmarked version of your revised paper without tracked changes. You should upload this as a separate file labeled 'Manuscript'.

If you would like to make changes to your financial disclosure, please include your updated statement in your cover letter. Guidelines for resubmitting your figure files are available below the reviewer comments at the end of this letter.

If applicable, we recommend that you deposit your laboratory protocols in protocols.io to enhance the reproducibility of your results. Protocols.io assigns your protocol its own identifier (DOI) so that it can be cited independently in the future. For instructions see: https://journals.plos.org/plosone/s/submission-guidelines#loc-laboratory-protocols. Additionally, PLOS ONE offers an option for publishing peer-reviewed Lab Protocol articles, which describe protocols hosted on protocols.io. Read more information on sharing protocols at https://plos.org/protocols?utm_medium=editorial-email&utm_source=authorletters&utm_campaign=protocols.

We look forward to receiving your revised manuscript.

Kind regards,

Ciro Clemente De Falco

Academic Editor

PLOS One

Journal Requirements:

If the reviewer comments include a recommendation to cite specific previously published works, please review and evaluate these publications to determine whether they are relevant and should be cited. There is no requirement to cite these works unless the editor has indicated otherwise.

Additional Editor Comments:

After reviewing the revised manuscript, the reviewers indicate that some additional revisions are still needed. These are more limited in scope compared to the previous round, but necessary to further strengthen the paper. I kindly invite you to address them and submit a revised version.

[Note: HTML markup is below. Please do not edit.]

Reviewers' comments:

Reviewer's Responses to Questions

**Comments to the Author**

1. If the authors have adequately addressed your comments raised in a previous round of review and you feel that this manuscript is now acceptable for publication, you may indicate that here to bypass the “Comments to the Author” section, enter your conflict of interest statement in the “Confidential to Editor” section, and submit your "Accept" recommendation.

Reviewer #1: All comments have been addressed

Reviewer #3: All comments have been addressed

**********

2. Is the manuscript technically sound, and do the data support the conclusions?

The manuscript must describe a technically sound piece of scientific research with data that supports the conclusions. Experiments must have been conducted rigorously, with appropriate controls, replication, and sample sizes. The conclusions must be drawn appropriately based on the data presented.

Reviewer #1: Yes

Reviewer #3: Partly

**********

3. Has the statistical analysis been performed appropriately and rigorously?

Reviewer #1: Yes

Reviewer #3: I Don't Know

**********

4. Have the authors made all data underlying the findings in their manuscript fully available?

The PLOS Data policy requires authors to make all data underlying the findings described in their manuscript fully available without restriction, with rare exception (please refer to the Data Availability Statement in the manuscript PDF file). The data should be provided as part of the manuscript or its supporting information, or deposited to a public repository. For example, in addition to summary statistics, the data points behind means, medians and variance measures should be available. If there are restrictions on publicly sharing data—e.g. participant privacy or use of data from a third party—those must be specified.

Reviewer #1: No

Reviewer #3: No

**********

5. Is the manuscript presented in an intelligible fashion and written in standard English?

PLOS ONE does not copyedit accepted manuscripts, so the language in submitted articles must be clear, correct, and unambiguous. Any typographical or grammatical errors should be corrected at revision, so please note any specific errors here.

Reviewer #1: Yes

Reviewer #3: No

**********

6. Review Comments to the Author

Please use the space provided to explain your answers to the questions above. You may also include additional comments for the author, including concerns about dual publication, research ethics, or publication ethics. (Please upload your review as an attachment if it exceeds 20,000 characters)

Reviewer #1: The authors have made a substantial effort in revising the manuscript. All the comments I previously raised have been addressed, and the manuscript is now much clearer, better structured, and more coherent.

There are, however, two aspects that would still benefit from further refinement.

First, the descriptive section on the BERT model remains relatively long and overly technical. I suggest focusing more specifically on what is necessary for understanding the study, as this would improve readability.

Second, although the Conclusions section is now more articulated, it could be strengthened by expanding the epistemological reflection on what it means for AI models to learn from divergent human classifications. Additionally, the brief reference to cybersecurity appears somewhat disconnected from the rest of the article; clarifying its relevance or removing it would enhance the coherence of the final section.

Overall, the manuscript has improved significantly.

Reviewer #3: Dear authors,

To have your paper published, further work is needed:

1. The study significance, research gap, research design, research questions are not there in the abstract and the Key words are missing.

2. The introduction section needs to be reconsidered in the sense of forgrounding the study significance, specifying the gap and ending with the research question. The last part of it about structing the article is out od lace, please remove it.

3. The APA system of writing is not consistently used throughout the paper.

4. The title 'Theoretical Framework' should be replaced with 'Literature Review' and should be reconsidered to have subsections.

5. There are many language problems such as the sentence immediately before Table 2.

6. I is preferable to have 'Analysis' instead of 'Preliminary analyses' where steps 1 ad 2 are replaced with 'Research question1' and 'Research question 2' to to better correlate the questions to the analysis section.

7. The discussion section should apear as 'Discussion' rather than concluding discussion.

8. Having short paragraphs is disctructing specially one- sentence or two-sentence paragraphs. Somtimes ideas need have a page to be fully addressed.

9. The 'Conclusion' section is missing. And it is better to have a section titled 'Limitations and suggestions' where the authors identify their study limitations followed by suggestions for further future studies.

10. The list of references should be reconsidered to correctly use the system of writing followed in the study.

**********

7. PLOS authors have the option to publish the peer review history of their article (what does this mean?). If published, this will include your full peer review and any attached files.

If you choose “no”, your identity will remain anonymous but your review may still be made public.

**Do you want your identity to be public for this peer review?**   For information about this choice, including consent withdrawal, please see our Privacy Policy.

Reviewer #1: No

Reviewer #3: **Yes:**  Nawal Fadhil Abbas

**********

[NOTE: If reviewer comments were submitted as an attachment file, they will be attached to this email and accessible via the submission site. Please log into your account, locate the manuscript record, and check for the action link "View Attachments". If this link does not appear, there are no attachment files.]

To ensure your figures meet our technical requirements, please review our figure guidelines: https://journals.plos.org/plosone/s/figures

You may also use PLOS’s free figure tool, NAAS, to help you prepare publication quality figures: https://journals.plos.org/plosone/s/figures#loc-tools-for-figure-preparation.

NAAS will assess whether your figures meet our technical requirements by comparing each figure against our figure specifications.

# Author comment: Author response to Decision Letter 2

**DOI:** 10.1371/journal.pone.0351336.r004

16 Jan 2026

Additional Editor Comments:

After reviewing the revised manuscript, the reviewers indicate that some additional revisions are still needed. These are more limited in scope compared to the previous round, but necessary to further strengthen the paper. I kindly invite you to address them and submit a revised version.

[Note: HTML markup is below. Please do not edit.]

Reviewers' comments:

Reviewer's Responses to Questions

Comments to the Author

1. If the authors have adequately addressed your comments raised in a previous round of review and you feel that this manuscript is now acceptable for publication, you may indicate that here to bypass the “Comments to the Author” section, enter your conflict of interest statement in the “Confidential to Editor” section, and submit your "Accept" recommendation.

Reviewer #1: All comments have been addressed

Reviewer #3: All comments have been addressed

2. Is the manuscript technically sound, and do the data support the conclusions?

The manuscript must describe a technically sound piece of scientific research with data that supports the conclusions. Experiments must have been conducted rigorously, with appropriate controls, replication, and sample sizes. The conclusions must be drawn appropriately based on the data presented.

Reviewer #1: Yes

Reviewer #3: Partly

3. Has the statistical analysis been performed appropriately and rigorously?

Reviewer #1: Yes

Reviewer #3: I Don't Know

4. Have the authors made all data underlying the findings in their manuscript fully available?

The PLOS Data policy requires authors to make all data underlying the findings described in their manuscript fully available without restriction, with rare exception (please refer to the Data Availability Statement in the manuscript PDF file). The data should be provided as part of the manuscript or its supporting information, or deposited to a public repository. For example, in addition to summary statistics, the data points behind means, medians and variance measures should be available. If there are restrictions on publicly sharing data—e.g. participant privacy or use of data from a third party—those must be specified.

Reviewer #1: No

Reviewer #3: No

5. Is the manuscript presented in an intelligible fashion and written in standard English?

PLOS ONE does not copyedit accepted manuscripts, so the language in submitted articles must be clear, correct, and unambiguous. Any typographical or grammatical errors should be corrected at revision, so please note any specific errors here.

Reviewer #1: Yes

Reviewer #3: No

6. Review Comments to the Author

Please use the space provided to explain your answers to the questions above. You may also include additional comments for the author, including concerns about dual publication, research ethics, or publication ethics. (Please upload your review as an attachment if it exceeds 20,000 characters)

Reviewer #1: The authors have made a substantial effort in revising the manuscript. All the comments I previously raised have been addressed, and the manuscript is now much clearer, better structured, and more coherent.

There are, however, two aspects that would still benefit from further refinement.

First, the descriptive section on the BERT model remains relatively long and overly technical. I suggest focusing more specifically on what is necessary for understanding the study, as this would improve readability.

Response: Thank you for the suggestion; we have further reduced the section on BERT models, focusing instead on their ability to be adapted to a specific context through fine-tuning on labeled data. This aspect is particularly relevant in our context, as researchers can manually label a subset of the data, thereby enabling the model to leverage this additional domain knowledge to classify large volumes of text. The revised text is as follows:

“To enhance the automatic classification and create a model specifically trained with the researchers' classification data, we decided to apply a Bidirectional Encoder Representations from Transformers (BERT) model pre-trained in Italian (More information about the model is available here: https://huggingface.co/dbmdz/bert-base-italian-cased). This model was fine-tuned using the researchers' classification results. BERT (Bidirectional Encoder Representations from Transformers) models are particularly well-suited for text classification tasks for several reasons. Since their introduction in 2018 (Devlin et al., 2019), and have been proved to be among the most performant Large Language Models (LLMs) in the literature. BERT models can be fine-tuned on task-specific data, allowing them to be adapted to a particular analytical context; this is especially useful in our study, where the model is trained on classifications provided by researchers to align its predictions with domain-specific interpretative criteria. This approach not only enhances the reliability of automatic text classification but also reduces the need for extensive manual classification efforts, therefore aligning well with our research objectives.”

Second, although the Conclusions section is now more articulated, it could be strengthened by expanding the epistemological reflection on what it means for AI models to learn from divergent human classifications. Additionally, the brief reference to cybersecurity appears somewhat disconnected from the rest of the article; clarifying its relevance or removing it would enhance the coherence of the final section.

Response:

Thank you for this question, which allows us to clarify how these models can be situated within the context of text classification. We have added the following text:

“From a methodological and epistemological perspective, our analysis is grounded in the distinction between two complementary evaluation dimensions.

The first concerns model accuracy, which is primarily related to the fine-tuning process and can be interpreted as a technical performance measure, reflecting how effectively the model learns from labeled data.

The second dimension concerns the degree of agreement, both among human researchers and between researchers and AI models. In this case, misalignment should not be interpreted as a failure of the model, but rather as an informative result.

The introduction of an AI model is not intended to impose an objective or definitive interpretive framework—something that is neither feasible nor desirable in the context of semantically ambiguous sociological data. Instead, the goal is for the model to reproduce a level of variability comparable to that observed among human coders. In this sense, disagreement is not an error to be eliminated, but a characteristic of the classification task itself.

An AI model can therefore be considered suitable for large-scale data processing insofar as it does not diverge from human classifications in a substantially different way than researchers already diverge from one another. Under this perspective, fine-tuned models do not replace human interpretation, but align with it, functioning as scalable extensions of human classificatory practices rather than as arbiters of a single “correct” classification.”

The reference to cybersecurity is indeed of interest to us, particularly given the recent advances in AI-based classification systems for anomaly detection. However, we acknowledge that addressing this topic in a sufficiently rigorous and comprehensible manner would risk diverting attention from the main focus of the paper. For this reason, we have decided to remove the reference to cybersecurity in order to improve the coherence and clarity of the section.

Overall, the manuscript has improved significantly.

Reviewer #3: Dear authors,

To have your paper published, further work is needed:

1. The study significance, research gap, research design, research questions are not there in the abstract and the Key words are missing.

Response: Thank you for this comment. The abstract has been reformulated to explicitly address the study significance, research gap, research design, and research questions, and

keywords have been added accordingly.

2. The introduction section needs to be reconsidered in the sense of forgrounding the study significance, specifying the gap and ending with the research question. The last part of it about structing the article is out od lace, please remove it.

Response: Thank you for this comment. The Introduction has been partially revised, as highlighted in the manuscript, in order to better foreground the study significance, specify the research gap, and clearly state the research questions. In addition, the final part describing the structure of the article has been removed, as requested.

3. The APA system of writing is not consistently used throughout the paper.

Response: Thank you for this comment. We have revised and standardized the references to ensure consistent use of the citation style throughout the paper.

4. The title 'Theoretical Framework' should be replaced with 'Literature Review' and should be reconsidered to have subsections.

Response:

Thank you for the suggestion. We have renamed the section “Literature Review” and reorganized it into the following subsections:

• Social networks as platforms

• Social media in public communication

• Public institutional communication

• Manual and automated post classification

5. There are many language problems such as the sentence immediately before Table 2.

Response: Thank you, we have carefully reviewed the English and corrected some typos.

6. I is preferable to have 'Analysis' instead of 'Preliminary analyses' where steps 1 ad 2 are replaced with 'Research question1' and 'Research question 2' to to better correlate the questions to the analysis section.

Response: Thank you. We have modified the section titles as suggested.

7. The discussion section should apear as 'Discussion' rather than concluding discussion.

Response: Thank you for the suggestion. We have expanded the discussion, also in response to the requests of Reviewer 1, and have therefore separated the Discussion from the Conclusions as suggested.

8. Having short paragraphs is disctructing specially one- sentence or two-sentence paragraphs. Somtimes ideas need have a page to be fully addressed.

Response: Thank you for this observation. While we recognize that some arguments may require extended discussion, the structure of the paper reflects common conventions in scientific writing, where clarity and conciseness are prioritized due to space limitations. Short paragraphs were used to emphasize specific points and to avoid conflating distinct aspects of the analysis.

9. The 'Conclusion' section is missing. And it is better to have a section titled 'Limitations and suggestions' where the authors identify their study limitations followed by suggestions for further future studies.

Response: Thank you for the suggestion. As mentioned above, we have separated the Discussion from the Conclusion. Regarding the limitations of the study, we chose to address them within the Discussion section, as we believe this approach supports a more cohesive interpretation of the results and helps readers better understand the scope and implications of each finding.

10. The list of references should be reconsidered to correctly use the system of writing followed in the study.

Response: Thank you for this comment. We have carefully revised the list of references and standardized it according to a consistent citation style used throughout the manuscript.

7. PLOS authors have the option to publish the peer review history of their article (what does this mean?). If published, this will include your full peer review and any attached files.

If you choose “no”, your identity will remain anonymous but your review may still be made public.

Do you want your identity to be public for this peer review? For information about this choice, including consent withdrawal, please see our Privacy Policy.

Reviewer #1: No

Reviewer #3: Yes: Nawal Fadhil Abbas

Attachment Submitted filename: Response to reviewers round 2.docx

# Peer review: Decision Letter 2

**DOI:** 10.1371/journal.pone.0351336.r005

**Authors:** Ciro Clemente De Falco

8 Apr 2026

PONE-D-25-21922R2

AI and Social Science. Automatic Classification Tools for Big Data Analysis in Sociological Research

PLOS One

Dear Dr. Nucita,

Thank you for submitting your manuscript to PLOS ONE. After careful consideration, we feel that it has merit but does not fully meet PLOS ONE’s publication criteria as it currently stands. Therefore, we invite you to submit a revised version of the manuscript that addresses the points raised during the review process.

Please submit your revised manuscript by May 23 2026 11:59PM. If you will need more time than this to complete your revisions, please reply to this message or contact the journal office at plosone@plos.org. When you're ready to submit your revision, log on to https://www.editorialmanager.com/pone/ and select the 'Submissions Needing Revision' folder to locate your manuscript file.

Please include the following items when submitting your revised manuscript:

- A letter that responds to each point raised by the academic editor and reviewer(s). You should upload this letter as a separate file labeled 'Response to Reviewers'.
- A marked-up copy of your manuscript that highlights changes made to the original version. You should upload this as a separate file labeled 'Revised Manuscript with Track Changes'.
- An unmarked version of your revised paper without tracked changes. You should upload this as a separate file labeled 'Manuscript'.

If you would like to make changes to your financial disclosure, please include your updated statement in your cover letter. Guidelines for resubmitting your figure files are available below the reviewer comments at the end of this letter.

If applicable, we recommend that you deposit your laboratory protocols in protocols.io to enhance the reproducibility of your results. Protocols.io assigns your protocol its own identifier (DOI) so that it can be cited independently in the future. For instructions see: https://journals.plos.org/plosone/s/submission-guidelines#loc-laboratory-protocols. Additionally, PLOS ONE offers an option for publishing peer-reviewed Lab Protocol articles, which describe protocols hosted on protocols.io. Read more information on sharing protocols at https://plos.org/protocols?utm_medium=editorial-email&utm_source=authorletters&utm_campaign=protocols.

As the corresponding author, your ORCID iD is verified in the submission system and will appear in the published article. PLOS supports the use of ORCID, and we encourage all coauthors to register for an ORCID iD and use it as well. Please encourage your coauthors to verify their ORCID iD within the submission system before final acceptance, as unverified ORCID iDs will not appear in the published article. *Only*   the individual author can complete the verification step; PLOS staff *cannot*   verify ORCID iDs on behalf of authors.

We look forward to receiving your revised manuscript.

Kind regards,

Ciro Clemente De Falco

Academic Editor

PLOS One

Journal Requirements:

1. If the reviewer comments include a recommendation to cite specific previously published works, please review and evaluate these publications to determine whether they are relevant and should be cited. There is no requirement to cite these works unless the editor has indicated otherwise.

2. Please review your reference list to ensure that it is complete and correct. If you have cited papers that have been retracted, please include the rationale for doing so in the manuscript text, or remove these references and replace them with relevant current references. Any changes to the reference list should be mentioned in the rebuttal letter that accompanies your revised manuscript. If you need to cite a retracted article, indicate the article’s retracted status in the References list and also include a citation and full reference for the retraction notice.

Additional Editor Comments:

Dear Authors,

the reviewers find the manuscript improved, but some revisions are still required before it can be accepted for publication.

Kind regards,

[Note: HTML markup is below. Please do not edit.]

Reviewers' comments:

Reviewer's Responses to Questions

**Comments to the Author**

1. If the authors have adequately addressed your comments raised in a previous round of review and you feel that this manuscript is now acceptable for publication, you may indicate that here to bypass the “Comments to the Author” section, enter your conflict of interest statement in the “Confidential to Editor” section, and submit your "Accept" recommendation.

Reviewer #1: All comments have been addressed

Reviewer #4: All comments have been addressed

**********

2. Is the manuscript technically sound, and do the data support the conclusions?

The manuscript must describe a technically sound piece of scientific research with data that supports the conclusions. Experiments must have been conducted rigorously, with appropriate controls, replication, and sample sizes. The conclusions must be drawn appropriately based on the data presented.

Reviewer #1: Yes

Reviewer #4: Yes

**********

3. Has the statistical analysis been performed appropriately and rigorously?

Reviewer #1: Yes

Reviewer #4: No

**********

4. Have the authors made all data underlying the findings in their manuscript fully available?

The PLOS Data policy requires authors to make all data underlying the findings described in their manuscript fully available without restriction, with rare exception (please refer to the Data Availability Statement in the manuscript PDF file). The data should be provided as part of the manuscript or its supporting information, or deposited to a public repository. For example, in addition to summary statistics, the data points behind means, medians and variance measures should be available. If there are restrictions on publicly sharing data—e.g. participant privacy or use of data from a third party—those must be specified.

Reviewer #1: Yes

Reviewer #4: No

**********

5. Is the manuscript presented in an intelligible fashion and written in standard English?

PLOS ONE does not copyedit accepted manuscripts, so the language in submitted articles must be clear, correct, and unambiguous. Any typographical or grammatical errors should be corrected at revision, so please note any specific errors here.

Reviewer #1: Yes

Reviewer #4: No

**********

6. Review Comments to the Author

Please use the space provided to explain your answers to the questions above. You may also include additional comments for the author, including concerns about dual publication, research ethics, or publication ethics. (Please upload your review as an attachment if it exceeds 20,000 characters)

Reviewer #1: In my opinion, all the comments raised in the previous rounds of review have been satisfactorily addressed.

Reviewer #4: The manuscript has clearly improved in structure, clarity, and overall coherence compared to the previous version. However, some aspects still require further refinement before publication. In particular, the data availability statement should be strengthened to clarify more explicitly what data are accessible and to what extent the analysis can be replicated, in line with journal requirements. The description of the statistical approach would also benefit from greater transparency, especially regarding the measures of agreement used and their interpretation. Additionally, the manuscript still presents some language issues, with sentences that are occasionally overly complex or not fully fluent, and would benefit from careful editing. Finally, the introduction and the section on BERT could be further streamlined to improve focus and readability, and the study's limitations should be articulated more clearly, ideally in a more visible, structured way.

**********

7. PLOS authors have the option to publish the peer review history of their article (what does this mean?). If published, this will include your full peer review and any attached files.

If you choose “no”, your identity will remain anonymous but your review may still be made public.

**Do you want your identity to be public for this peer review?**   For information about this choice, including consent withdrawal, please see our Privacy Policy.

Reviewer #1: No

Reviewer #4: No

**********

[NOTE: If reviewer comments were submitted as an attachment file, they will be attached to this email and accessible via the submission site. Please log into your account, locate the manuscript record, and check for the action link "View Attachments". If this link does not appear, there are no attachment files.]

To ensure your figures meet our technical requirements, please review our figure guidelines: https://journals.plos.org/plosone/s/figures

You may also use PLOS’s free figure tool, NAAS, to help you prepare publication quality figures: https://journals.plos.org/plosone/s/figures#loc-tools-for-figure-preparation.

NAAS will assess whether your figures meet our technical requirements by comparing each figure against our figure specifications.

# Author comment: Author response to Decision Letter 3

**DOI:** 10.1371/journal.pone.0351336.r006

23 Apr 2026

Additional Editor Comments:

Dear Authors,

the reviewers find the manuscript improved, but some revisions are still required before it can be accepted for publication.

Kind regards,

[Note: HTML markup is below. Please do not edit.]

Reviewers' comments:

Reviewer's Responses to Questions

Comments to the Author

1. If the authors have adequately addressed your comments raised in a previous round of review and you feel that this manuscript is now acceptable for publication, you may indicate that here to bypass the “Comments to the Author” section, enter your conflict of interest statement in the “Confidential to Editor” section, and submit your "Accept" recommendation.

Reviewer #1: All comments have been addressed

Reviewer #4: All comments have been addressed

2. Is the manuscript technically sound, and do the data support the conclusions?

The manuscript must describe a technically sound piece of scientific research with data that supports the conclusions. Experiments must have been conducted rigorously, with appropriate controls, replication, and sample sizes. The conclusions must be drawn appropriately based on the data presented.

Reviewer #1: Yes

Reviewer #4: Yes

3. Has the statistical analysis been performed appropriately and rigorously?

Reviewer #1: Yes

Reviewer #4: No

4. Have the authors made all data underlying the findings in their manuscript fully available?

The PLOS Data policy requires authors to make all data underlying the findings described in their manuscript fully available without restriction, with rare exception (please refer to the Data Availability Statement in the manuscript PDF file). The data should be provided as part of the manuscript or its supporting information, or deposited to a public repository. For example, in addition to summary statistics, the data points behind means, medians and variance measures should be available. If there are restrictions on publicly sharing data—e.g. participant privacy or use of data from a third party—those must be specified.

Reviewer #1: Yes

Reviewer #4: No

5. Is the manuscript presented in an intelligible fashion and written in standard English?

PLOS ONE does not copyedit accepted manuscripts, so the language in submitted articles must be clear, correct, and unambiguous. Any typographical or grammatical errors should be corrected at revision, so please note any specific errors here.

Reviewer #1: Yes

Reviewer #4: No

6. Review Comments to the Author

Please use the space provided to explain your answers to the questions above. You may also include additional comments for the author, including concerns about dual publication, research ethics, or publication ethics. (Please upload your review as an attachment if it exceeds 20,000 characters)

Reviewer #1: In my opinion, all the comments raised in the previous rounds of review have been satisfactorily addressed.

Reviewer #4: The manuscript has clearly improved in structure, clarity, and overall coherence compared to the previous version. However, some aspects still require further refinement before publication. In particular, the data availability statement should be strengthened to clarify more explicitly what data are accessible and to what extent the analysis can be replicated, in line with journal requirements.

Response: Thank you for your comment. We have updated the Data Availability statement to more clearly specify which data are publicly available and to explicitly address the replicability of the analysis.

Revised Data Availability Statement

The raw data (i.e., Facebook posts from the official pages of the University of Messina and the University of Salerno) cannot be publicly shared due to Meta’s data usage policies, which prohibit the redistribution of content extracted from the platform.

However, the annotated datasets and trained models used for AI model development are publicly available on our Hugging Face repository:

https://huggingface.co/HuM-HILab/pub_comm_classification

These resources include the full annotation schema, data structure, and model configurations used in the study, and are intended to enable transparency and reproducibility of the analytical workflow. While the original post texts are not included, the shared materials provide all necessary components to replicate the methodology.

Researchers who have independent access to comparable raw data (i.e., post texts) can reproduce the analysis using the provided annotations and models, or apply the same pipeline to alternative datasets. For further details on the data collection and processing procedures, researchers may contact the HuM-HI Laboratory at humhilab@unime.it or visit humhilab.unime.it.

The description of the statistical approach would also benefit from greater transparency, especially regarding the measures of agreement used and their interpretation.

Response: Thank you for your comment. We have expanded the methodological section to clarify the rationale for selecting Fleiss’ Kappa, highlighting its appropriateness for multi-rater agreement and its distinction from Cohen’s Kappa.

Additionally, the manuscript still presents some language issues, with sentences that are occasionally overly complex or not fully fluent, and would benefit from careful editing.

Response: Thank you for your comment. We have carefully revised the entire manuscript, paying particular attention to improving clarity and fluency, and simplifying sentences that were overly complex or unclear.

Finally, the introduction and the section on BERT could be further streamlined to improve focus and readability,

Response: Thank you for your comment. We have made revisions where possible to improve the clarity and flow of both the Introduction and the section on BERT, while retaining the key references and information needed for a proper understanding of the research.

and the study's limitations should be articulated more clearly, ideally in a more visible, structured way.

Response: Thank you for your comment. We have revised this section to more clearly articulate the study’s limitations, organizing them in a dedicated and structured way. We have also clarified the role of the empirical context, emphasizing that while it may limit generalizability, the primary contribution of the study is methodological.

7. PLOS authors have the option to publish the peer review history of their article (what does this mean?). If published, this will include your full peer review and any attached files.

If you choose “no”, your identity will remain anonymous but your review may still be made public.

Do you want your identity to be public for this peer review? For information about this choice, including consent withdrawal, please see our Privacy Policy.

Reviewer #1: No

Reviewer #4: No

Attachment Submitted filename: Response to reviewers round 3.docx

# Peer review: Decision Letter 3

**DOI:** 10.1371/journal.pone.0351336.r007

**Authors:** Ciro Clemente De Falco

26 May 2026

AI and Social Science. Automatic Classification Tools for Big Data Analysis in Sociological Research

PONE-D-25-21922R3

Dear Dr. Nucita,

We’re pleased to inform you that your manuscript has been judged scientifically suitable for publication and will be formally accepted for publication once it meets all outstanding technical requirements.

Within one week, you’ll receive an e-mail detailing the required amendments. When these have been addressed, you’ll receive a formal acceptance letter and your manuscript will be scheduled for publication.

An invoice will be generated when your article is formally accepted. Please note, if your institution has a publishing partnership with PLOS and your article meets the relevant criteria, all or part of your publication costs will be covered. Please make sure your user information is up-to-date by logging into Editorial Manager at Editorial Manager® and clicking the ‘Update My Information' link at the top of the page. For questions related to billing, please contact billing support.

If your institution or institutions have a press office, please notify them about your upcoming paper to help maximize its impact. If they’ll be preparing press materials, please inform our press team as soon as possible -- no later than 48 hours after receiving the formal acceptance. Your manuscript will remain under strict press embargo until 2 pm Eastern Time on the date of publication. For more information, please contact onepress@plos.org.

Kind regards,

Ciro Clemente De Falco

Academic Editor

PLOS One

Additional Editor Comments (optional):

Dear Author,

I am pleased to inform you that your manuscript has been positively evaluated in its revised version and is now considered suitable for publication.

The reviewers appreciated the significant improvements made compared to the previous version. In particular, they noted that the revisions concerning methodological transparency, data availability, clarification of the agreement measures, and the restructuring of the limitations section have strengthened the overall quality, clarity, and coherence of the paper.

The manuscript now presents a clearer and more rigorous methodological contribution regarding the use of AI-assisted classification tools in sociological research.

Best regards,

Reviewers' comments:

Reviewer's Responses to Questions

**Comments to the Author**

1. If the authors have adequately addressed your comments raised in a previous round of review and you feel that this manuscript is now acceptable for publication, you may indicate that here to bypass the “Comments to the Author” section, enter your conflict of interest statement in the “Confidential to Editor” section, and submit your "Accept" recommendation.

Reviewer #4: All comments have been addressed

**********

2. Is the manuscript technically sound, and do the data support the conclusions?

The manuscript must describe a technically sound piece of scientific research with data that supports the conclusions. Experiments must have been conducted rigorously, with appropriate controls, replication, and sample sizes. The conclusions must be drawn appropriately based on the data presented.

Reviewer #4: Yes

**********

3. Has the statistical analysis been performed appropriately and rigorously?

Reviewer #4: Yes

**********

4. Have the authors made all data underlying the findings in their manuscript fully available?

The PLOS Data policy requires authors to make all data underlying the findings described in their manuscript fully available without restriction, with rare exception (please refer to the Data Availability Statement in the manuscript PDF file). The data should be provided as part of the manuscript or its supporting information, or deposited to a public repository. For example, in addition to summary statistics, the data points behind means, medians and variance measures should be available. If there are restrictions on publicly sharing data—e.g. participant privacy or use of data from a third party—those must be specified.

Reviewer #4: Yes

**********

5. Is the manuscript presented in an intelligible fashion and written in standard English?

PLOS ONE does not copyedit accepted manuscripts, so the language in submitted articles must be clear, correct, and unambiguous. Any typographical or grammatical errors should be corrected at revision, so please note any specific errors here.

Reviewer #4: Yes

**********

6. Review Comments to the Author

Please use the space provided to explain your answers to the questions above. You may also include additional comments for the author, including concerns about dual publication, research ethics, or publication ethics. (Please upload your review as an attachment if it exceeds 20,000 characters)

Reviewer #4: The manuscript has improved significantly compared to the previous version, and the authors have addressed the main concerns raised during the review process in a satisfactory manner. In particular, the revisions related to methodological transparency, data availability, clarification of the agreement measures, and the restructuring of the limitations section have strengthened the overall quality and coherence of the paper.

The study now presents a clearer and more rigorous methodological contribution regarding the use of AI-assisted classification tools in sociological research. While a few minor language and formatting issues could still benefit from careful editorial proofreading, these do not affect the scientific quality of the manuscript.

The manuscript is now suitable for publication.

**********

7. PLOS authors have the option to publish the peer review history of their article (what does this mean?). If published, this will include your full peer review and any attached files.

If you choose “no”, your identity will remain anonymous but your review may still be made public.

**Do you want your identity to be public for this peer review?**   For information about this choice, including consent withdrawal, please see our Privacy Policy.

Reviewer #4: No

**********

# Editor report: Acceptance letter

**DOI:** 10.1371/journal.pone.0351336.r008

**Authors:** Ciro Clemente De Falco

PONE-D-25-21922R3

PLOS One

Dear Dr. Nucita,

I'm pleased to inform you that your manuscript has been deemed suitable for publication in PLOS One. Congratulations! Your manuscript is now being handed over to our production team.

At this stage, our production department will prepare your paper for publication. This includes ensuring the following:

* All references, tables, and figures are properly cited

* All relevant supporting information is included in the manuscript submission,

* There are no issues that prevent the paper from being properly typeset

You will receive further instructions from the production team, including instructions on how to review your proof when it is ready. Please keep in mind that we are working through a large volume of accepted articles, so please give us a few days to review your paper and let you know the next and final steps.

Lastly, if your institution or institutions have a press office, please let them know about your upcoming paper now to help maximize its impact. If they'll be preparing press materials, please inform our press team within the next 48 hours. Your manuscript will remain under strict press embargo until 2 pm Eastern Time on the date of publication. For more information, please contact onepress@plos.org.

You will receive an invoice from PLOS for your publication fee after your manuscript has reached the completed accept phase. If you receive an email requesting payment before acceptance or for any other service, this may be a phishing scheme. Learn how to identify phishing emails and protect your accounts at https://explore.plos.org/phishing.

If we can help with anything else, please email us at customercare@plos.org.

Thank you for submitting your work to PLOS ONE and supporting open access.

Kind regards,

PLOS ONE Editorial Office Staff

on behalf of

Dr. Ciro Clemente De Falco

Academic Editor

PLOS One
